# Reproduction and Development in Calcareous Sponges: A Panorama of the Last Two Centuries

**DOI:** 10.1002/mrd.70129

**Published:** 2026-07-06

**Authors:** Bruno Cajado, Emilio Lanna

**Affiliations:** ^1^ Graduate Program in Biodiversity and Evolution, Institute of Biology Federal University of Bahia Salvador Bahia Brazil; ^2^ Instituto de Biologia Universidade Federal da Bahia Salvador Bahia Brazil; ^3^ National Institute of Science and Technology in Interdisciplinary and Transdisciplinary Studies in Ecology and Evolution (INCT IN‐TREE) Salvador Bahia Brazil

**Keywords:** asexual reproduction, Calcarea, evolutionary developmental biology, gametogenesis, Porifera

## Abstract

Nearly 200 years ago, scientists began examining reproductive cells in calcareous sponges (Calcarea, Porifera). Since then, 238 studies have been published, encompassing descriptions of gametes and embryos (including ultrastructural studies), life history, asexual reproduction, regeneration, and molecular aspects of development. Here, we present a comprehensive synthesis of two centuries of research on the development of Calcarea and review the different steps of the reproductive processes within this class. We analyzed temporal trends, thematic foci, authors, and geographic distribution of the studied species, and the reproductive characteristics of both subclasses (Calcinea and Calcaronea). Bibliographic productivity increased in gradual waves, from classical histology through ultrastructural and experimental embryology to advanced live imaging and molecular approaches. The past half‐century has shown acceleration, yet with a persistent geographic bias toward the Mediterranean and North Atlantic species. Molecular datasets are beginning to link phylogenetic context and gene expression during development, but remain fragmentary and lack standardization within the class. We outline priorities for the coming years and identify critical unanswered questions. While significant challenges remain, confronting them actively advances research on Calcarea reproduction and development and firmly establishes it as a distinct field within sponge reproductive science.

## 1 Introduction

Two centuries ago, Grant (1826) referred to spaces between spicules “for the lodgment of the soft parts and ova of this animal.” But it was only after three decades that a description of larvae of a calcareous sponge (Calcarea, Porifera), *Sycon ciliatum*, appeared in the scientific literature (Lieberkühn 1859). Knowledge in sponge reproduction science (SRS) (Ereskovsky 2007, 2010; Lanna et al. 2018a) has expanded over the past two centuries with different focuses, including embryonic descriptions, ultrastructure of reproductive elements, life history, asexual reproduction, regeneration, and molecular development (Ereskovsky 2010; Lanna et al. 2018a). From Grant's early descriptions to current molecular evidence, the study of sponge reproduction mirrors the broader evolution of the biological sciences.

Calcarea is one of the four classes of Porifera and represents only approximately 8.5% of all extant Porifera species. It forms a clade with Homoscleromorpha, which is the sister group of Silicea (Demospongiae and Hexactinellida) (Wörheide et al. 2012). Calcarea and its two subclasses (Calcinea and Calcaronea) are monophyletic (Manuel et al. 2003); however, most families and genera are not, and their relationships are still debatable (Voigt et al. 2012; Alvizu et al. 2018; Klautau et al. 2020). Calcarea is the single class of sponges in which all six types of aquiferous systems have been described (Cavalcanti and Klautau 2011; Lopes and Klautau 2023), with four of them found exclusively in this class (i.e., asconoid, sylleibid, solenoid, and kladonoid). Another characteristic that distinguishes this group from other sponges is the presence of free spicules made of calcium carbonate (Manuel et al. 2003; Wörheide et al. 2012). The systematics of this group remains challenging, mostly because of the paucity of morphological characters available and the recurrent occurrence of convergent traits in different lineages (Voigt et al. 2012; Lopes and Klautau 2023). Interestingly, aspects of reproduction and development seem promising for understanding the evolution and systematics of the group. For example, among the few taxonomic characters available to distinguish the subclasses, the larval type is a well‐established criterion: Calcinea possess calciblastula‐type larvae, whereas Calcaronea exhibit amphiblastula‐type larvae (Hartman 1958; Manuel et al. 2003; Ereskovsky 2010; Maldonado and Wahab 2025). Some researchers dedicated part of their careers to investigating the unique traits of Calcarea over the decades, including Ernst Haeckel, Octave Duboscq, Odette Tuzet, Marie‐France Gallissian, Galina Korotkova, and others (Ereskovsky 2007, 2010). Nonetheless, Calcarea remains an interesting and promising model for investigating fundamental questions in developmental and reproductive biology.

Consequently, one would expect a prolific literature on the SRS of Calcarea. However, this group of sponges appears to have been historically neglected in SRS and evolutionary studies. One possible reason is their predominantly cryptic habitats, inconspicuous body size, and lack of vivid coloration, which make these sponges difficult to detect in the field, alongside geographic bias (Manuel and Le Parco 2000; Calazans and Lanna 2019; De Voogd et al. 2025). In addition, the relatively small number of specialists dedicated to calcareous sponges has likely contributed to the comparatively limited number of studies available for the group, compared to the other classes, such as Demospongiae. Consequently, important gaps persist regarding oocyte nutrition, embryonic inversion, and developmental variation within the class, despite more than a century of research since Haeckel's classical monograph (1872). These limitations also hinder the application of modern molecular approaches, including studies on gene repertoires, developmental pathways, cellular regulation, and interspecific variation. Nevertheless, it remains unclear whether the apparently “neglected” status of Calcarea reflects a genuine lack of investigation or methodological difficulties associated with accessing older literature, much of which is written in languages other than English (see Leys 2005). A comprehensive review of the literature on reproduction and development in Calcarea is therefore timely and relevant for identifying and contextualizing these longstanding gaps. Addressing the critical gaps that remain in the study of SRS in Calcarea is essential for establishing the current “state of the art” in this field. This includes assessing how many studies provide developmental data, identifying which investigations describe reproductive structures or experimentally examine asexual reproduction, and determining which species and geographic regions have been covered. It also requires evaluating publication trends over time and tracing the evolution of scientific understanding over the past two centuries. Furthermore, it is necessary to examine how molecular data have been integrated with morphological evidence. It would be possible to observe persistent gaps, indicating not only how they affect our understanding of Calcarea but also constrain broader evolutionary interpretations, as Porifera represents one of the earliest diverging lineages within Metazoa (McCarthy et al. 2023). A better understanding of the reproductive and developmental biology of Calcarea is critical for reconstructing ancestral traits of animals (Lanna et al. 2024).

Therefore, the aims of this work were to quantify and analyze published studies addressing the umbrella terms “development” and “reproduction” in Calcarea, encompassing topics such as morphology, gametogenesis, embryogenesis, molecular pathways, life history, asexual reproduction, regeneration, and evolutionary developmental biology (evo‐devo). The extensive list of literature compiled here, systematized by species, topic, and region of the globe, provides a solid scaffold for future research on the class. In addition to the quantitative analyses of the publications, we provide a review of various aspects of Calcarea's development. This review includes a historical perspective to illustrate how the current knowledge evolved through the history of Calcarea SRS. With both objectives in mind, this review provides a structured reference framework for any researcher seeking to study the reproduction and development of Calcarea.

## 2 Materials and Methods

To quantify the body of literature on Calcarea reproduction and development, including primary literature and review works, as articles, book chapters, entire books, and classical theses from the 19th and 20th centuries, we conducted an exhaustive literature compilation with quantitative descriptive analysis based on snowball sampling, initially based on previously published reviews (see Section 3, 3.2.3). In this study, an “entry” represents one species or morphotype reported in a single publication. Thus, one publication may contain multiple entries. Both the initial set of publications and the references cited within them were examined to capture nearly all relevant works on the topic.

Our review spans almost two centuries, from 1826 to December 31, 2024. We grouped the publications into four 50‐year intervals: I: 1826–1875; II: 1876–1925; III: 1926–1975; IV: 1976–2024, the last covering 49 years. To help clarify a small number of unresolved records, we contacted researchers working on Calcarea when additional information was required and scrutinized the reference lists of multiple publications. No restrictions were applied regarding language or publication date until 2024. We are aware of additional academic outputs, such as undergraduate and graduate theses, conference abstracts, and institutional reports, produced in different languages over time. However, these outputs were excluded as “gray literature” owing to limitations in peer review, accessibility, and standardization, which may compromise the reliability of the data. Thus, this study combines an extensive historical review with snowball citation tracking. All compiled data are available in four spreadsheets provided as Supplementary Material (Tables S1–S4). In addition, complete publications that have entered the public domain are available from the authors upon request.

Of all works detected using this technique, only 8 (3.4%) were inaccessible to us; whenever possible, data from secondary sources were incorporated into our analyses (Table S1). All entries were identified to the species level, with species names updated, when necessary, according to the current classification (De Voogd et al. 2025). However, eight entries were identified only to the genus level, so we retained the original genus names (Table S1). For our analysis, all works were retained, even those that only cited developmental traits.

## 3 Results and Discussion

### 3.1 Temporal Trends and Descriptions of the Publications

We identified 238 publications containing developmental information on Calcarea, including research articles, book chapters, reviews, and notes. Most of these works were written in English (143 works, 60.1%), followed by French (49), German (26), Russian (14), Italian (4), Croatian (1), and one in both English and French (Table S1). Of all publications, 192 works were classified as “Primary literature” on Calcarea (original figures, descriptions, experiments, or reports on reproduction; see Tables S1–S3), while 46 works were classified as “Reviews” addressing aspects of reproduction and development in Calcarea (Table S4). Since 1990, 64 primary literature addressing reproduction and/or development have been published, all written in English, with only two exceptions (Anakina and Drozdov 2000, in Russian; De Vos et al. 1991, also in French). The publications classified as *Reviews* were published predominantly in English (*n* = 35, 76.1%). From 1826 to 1920, all 51 primary literature works were single‐authored. By contrast, among the 51 most recent works (2000–2024), all had multiple authors (Table S1).

**Figure 1:**
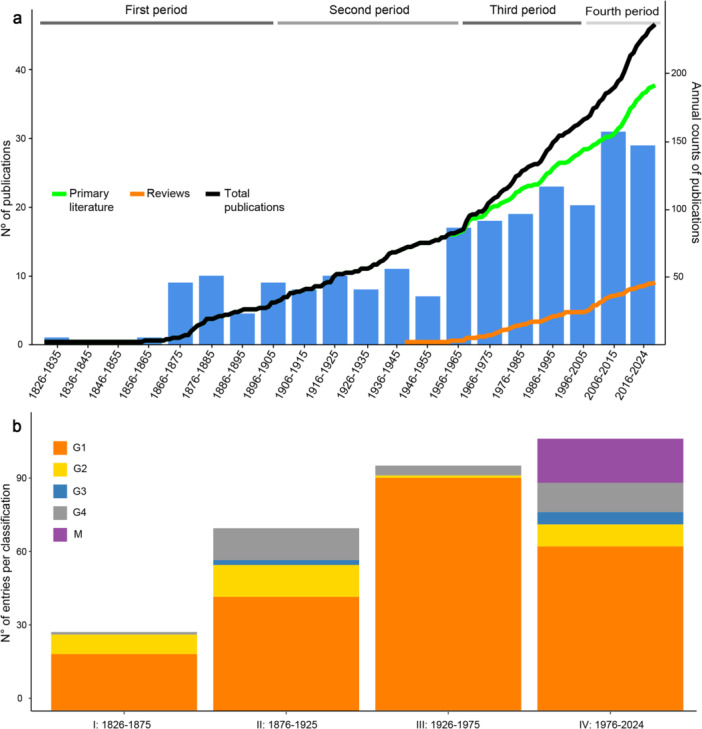
Quantitative trends of the studies on the reproduction and development of Calcarea (1826–2024). (a) Publications per decade (blue bars), with cumulative counts shown as a line (*n* = 238). The “periods” correspond to the different periods of sponge reproduction science (*sensu* Ereskovsky 2007). (b) Entries classifications for primary literature (*n* = 322, as a single article can generate multiple entries): G1—description with illustrations; G2—description only; G3—illustrations only; G4—citation only; M—molecular data (G0M, G1M, G3M, G4M), divided into intervals of 50 years. Note that the 50‐year intervals shown here do not coincide with the conceptual periods of sponge reproduction science.

Grouping these 238 publications into four 50‐year intervals, half of them (51.7%, *n* = 123) were published in the most recent period (IV), with the previous intervals showing a gradual increase in publications (I: 11; II: 43; III: 61). Noteworthy, the first “review” publication only appeared in period III (Brien 1943). These data reveal distinct growth phases, consistent with the segmented growth of scientific expansion over time (Bornmann and Mutz 2015). Indeed, in the Porifera literature (Ereskovsky 2010; Lanna et al. 2018a), there is an almost fourfold increase in publications from period I to II, and a twofold increase from period III to IV (Figure 1a). The publication record also reflects the broader exponential growth of scientific journals; for example, the first documented study on Calcarea development (1859) appeared nearly two centuries after the launch of the first scientific journal (1665; Ghasemi et al. 2022). Also, the nearly three‐decade interval between the first mention of a sponge ova (Grant 1825) and the published description of calcareous sponge larvae (Lieberkühn 1859), together with the accumulation of recent data, underscores the slow yet persistent progress of sponge biology.

#### 3.1.1 Reviews

We analyzed reviews separately from the primary literature, as they generally do not provide original data. The 46 identified reviews address Calcarea to varying extents, ranging from brief mentions to comprehensive treatments (Table S4). None of them focused solely on Calcarea development. Other major contributions include a comprehensive review of the biology of Calcarea (Tuzet 1973), and works centered on the classification of the group, such as Hartman (1958) on Bidder's system, Burton (1963) on all Calcarean species systematic, Borojevic et al. (1990) on Calcinea systematics, Borojevic et al. (2000) on Calcaronea systematics, and Manuel et al. (2003) on the monophyly of the subclasses. In these works, reproduction aspects are provided to support the systematics proposed by the authors. Most reviews (*n* = 28) considered the phylum Porifera as a whole, often emphasizing Demospongiae, with examples ranging from classic syntheses (e.g., Brien 1943; Korotkova 1963a) to recent updates (e.g., Ereskovsky et al. 2021; Lanna et al. 2024). Eleven reviews incorporated developmental data on Calcarea within a broader comparative framework (Porifera, non‐bilaterians, Metazoa, or even across kingdoms), while four of which (Maldonado 2004, 2014; Ereskovsky 2007; Wörheide et al. 2012) emphasized general supraspecific patterns. Several other works cited specific species while providing general overviews of subclasses (e.g., Fell 1974, 1989; Maldonado and Bergquist 2002; Leys and Ereskovsky 2006; Ereskovsky 2010; Degnan et al. 2015). Some papers do provide comprehensive reviews but also include primary data (e.g., Dendy 1914; Duboscq and Tuzet 1937b). Therefore, these works were classified under Section 3.1.2.

#### 3.1.2 Overview of the Primary Literature

Of the 192 primary literature works found, eight could not be accessed. From the dataset of 184 works to which we had full access, we classified the primary literature by the type of developmental information that was provided (Table 1). We created a category (G4) with only the citation of some developmental information, meaning that some feature of the reproduction or development of the species was present or was relevant to the subject of the study. This framework allows comparison between pre‐ and post‐molecular studies, while emphasizing the availability of genetic information. We found 322 entries within the 184 analyzed works. Most of the entries were classified as G1 (*n* = 235; 73.0%), with fewer in G4 (*n* = 33), G2 (*n* = 31), G3 (*n* = 7), and G0M (*n* = 8). All G1M (*n* = 5) and G3M (*n* = 3) were restricted to studies of *S. ciliatum* (Fortunato et al. 2012, 2014, 2015, 2016; Leininger et al. 2014; Bråte et al. 2015; Soubigou et al. 2020; Caglar et al. 2021) (Figure 1b). None were classified as G2M or G4M (Table S1). Overall, the dataset provides extensive morphological information but only limited molecular data (see Section 3.3).

**Table 1:** Classification codes used to categorize sources according to the type of information available on reproduction and development in Calcarea. Categories “G1–G4” represent different combinations of textual descriptions, illustrations, and citations, while the suffix “M” indicates the presence of associated molecular data. G0M denotes sources containing only molecular data. See Tables S1–S3 for details.

Category	Description	N° of entries
G1	Text description with illustrations	235
G2	Text description only	31
G3	Illustrations only	7
G4	Citation only	33
G0M	Presence of molecular data	8
G1M	G1 + molecular data	5
G3M	G3 + molecular data	3

**Figure 2:**
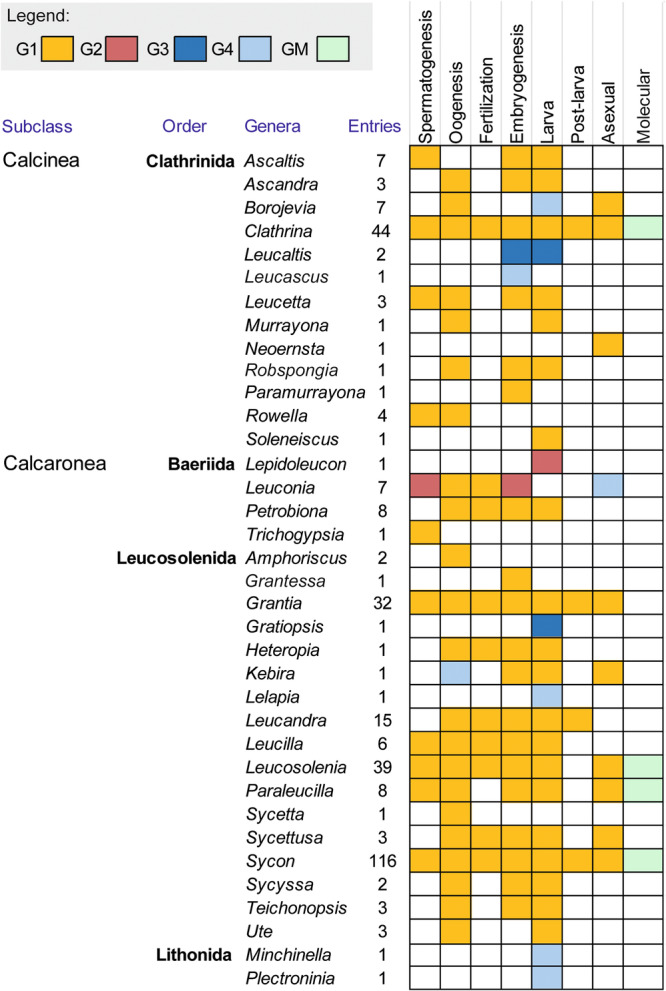
Summary of the different aspects of the reproduction and development of Calcarea genera (*n* = 322 of recorded entries). Characters: (1) spermatogenesis; (2) oogenesis; (3) fertilization (spermiocyst); (4) embryogenesis; (5) larva; (6) post‐larva; (7) asexual reproduction (including olynthus and regeneration); (8) molecular data related to development. Color‐coded boxes indicate the classification group: G1—description with illustrations; G2—description only; G3—illustrations only; G4—citation only; GM—molecular data (G0M, G1M, G3M). All references are listed in the Supporting Information S1: Supplementary Material. Complete datasets for all species and reproductive elements are provided in Table S1.

Eighty‐seven species of calcareous sponges have been studied at different levels of detail, corresponding to 36 genera (Table S2). When restricting to cases with both figures and descriptions (G1) of reproductive elements (i.e., spermatic cysts, oocytes, embryos, free and metamorphosing larvae), data are available for at least one element in 62 species of 29 genera (Figure 2). Yet, these numbers can mask essential discrepancies. For instance, 32 species were examined only once across the literature, though not necessarily within the same study (Table S2). Among these 62 species, 19 were studied exclusively in the 19th century (i.e., *Ascaltis poterium*, *Alcaltis depressa*, *Ascaltis wilsoni*, *Clathrina primordialis*, *Rowella haeckeliana*, *Amphoriscus oviparus*, *Amphoriscus testiparus*, *Grantessa intusarticulata*, *Leucandra ananas*, *Leucascus clavatus*, *Leucandra echinata*, *Leucilla echina*, *Leucilla uter*, *Sycetta primitiva*, *Sycon humboldti*, *Sycon quadrangulatum*, *Sycyssa huxleyi*, *Ute glabra*, and *Ute syconoides*). These cases highlight the need to revisit such species and their histological and cellular descriptions using modern technologies.

Traits related to asexual reproduction were reported in 44 works: regeneration (including primmorphs and fragmentation; 35 entries), post‐embryonic growth (including olynthus; 21), and asexual reproduction (8). Of these studies, 36 works did not describe or illustrate any sexual reproductive elements. Asexual reproduction was also incorporated into molecular data in three studies (Padua et al. 2016; Soubigou et al. 2020; Caglar et al. 2021), highlighting the current importance of asexual processes in Calcarea.

The phenology of the reproduction was documented in 51 works. Twenty‐four of which only recorded the presence of elements during a specific period (Table S1). Reported patterns include restriction to a single month (e.g., February; Miklucho‐Maclay 1868), occurrence in summer (e.g., Bianco 1888), for approximately half of the year (e.g., Orton 1920), after summer (Sarà 1955a), absence in winter (e.g., Gilis et al. 2011), or persistence year‐round (e.g., Lanna and Klautau 2015, 2018).

#### 3.1.3 Taxa Coverage of Sexual Reproduction in Calcarea

In total, 152 published works (79.2%) documented reproductive elements (categorized here as male elements, oocytes, fertilization, embryos, larvae, and post‐larvae) of 268 entries (83.2%) of 87 species (Table S1). Studies investigating multiple reproductive elements were found for 128 entries (38.7%). Oogenesis was reported for 136 entries; spermatogenesis and fertilization (spermiocysts), for 44 entries each; embryos, for 103; larvae, for 137; and post‐larvae, for 24 (Figure 2; Table S1). These results emphasize that female elements (oocytes, embryos, larvae) are easier to observe when compared to male gametes and fertilization (see below).

Within the subclass Calcinea, which comprises a single order (Clathrinida), we found 76 entries (22.9%), representing 31 species (11.9% of the currently accepted names) across 13 genera (out of 28) (Table S5). The most represented genus of Calcinea was *Clathrina* (*n* = 44, 13.3% of total entries), with *C. coriacea* (*n* = 15) being the most studied species (Table S2). All of these species had at least one work with developmental descriptions and/or illustrations, except *Rowella simplicissima* and *Rowella vera*, whose oocytes were only cited by Lopes and Klautau (2023), and *Leucascus clavatus*' embryos by Dendy (1893; Tables S1). A full list of species and their respective publications is provided in Table S3. To date, no molecular data on development are available for this subclass (except for population genetics; Padua et al. 2018).

The subclass Calcaronea was more extensively studied, comprising 255 entries (77.0%) across its three orders: Baeriida (*n* = 17), Leucosolenida (*n* = 235), but no reproductive data are available for the order Lithonida, apart from citations of larval type (*n* = 2). Calcaronea with developmental studies covered 23 out of 58 genera (39.7%) and 56 out of 575 species (ca. 10%). Proportionally, due to the large number of Leucosolenida species (*n* = 535, 64.1% of all calcareous sponge species), this order was the least studied, with only 8.9% (*n* = 48) of its species examined, despite representing 64.1% of all Calcarea entries and 93.0% of all Calcaronea entries. The most represented genus was *Sycon* (*n* = 116, 35.0%), and the most studied species were *S. raphanus* (*n* = 43), *S. ciliatum* (*n* = 32), and *Grantia compressa* (*n* = 31). Forty‐eight species had at least one work reporting reproductive or developmental descriptions and/or illustrations (Table S2). Exceptions included *Grantia capillosa*, *Lelapia australis*, *Sycon frustulosum*, *Sycon scaldiense*, and the two Lithonida (*Minchinella* sp. and *Plectroninia tecta*; Vacelet 1991), to which the reproductive elements were only cited (Table S2), as well as *Leucosolenia corallorrhiza* and *Leucosolenia eleanor*, for which only molecular data are available in the literature (Bebenek et al. 2004; Lavrov et al. 2024). Morphological and molecular developmental traits are documented only for four species (*Leucosolenia complicata*, *Sycon raphanus*, *Sycon coactum*, and notably *Sycon ciliatum*—with 9 of the 18 molecular entries), all restricted to temperate/polar regions and representing just two genera within a single order. This highlights the limited generalizability of molecular developmental data across Calcarea.

Even though the total number of calcareous sponge species that had reproductive and developmental patterns examined seems significant, 87 spp. (ca. 10.4% of all Calcarea), the number of species that have had all developmental stages (gametogenesis, embryogenesis, and larva) studied is much more limited (12 species: *Clathrina blanca*, *Clathrina coriacea*, *Clathrina primordialis*, *Grantia compressa*, *Leucetta giribeti*, *L. complicata*, *Paraleucilla magna*, *S. ciliatum*, *S. coactum*, *S. raphanus*, *Sycon sycandra*, and *Sycettusa hastifera*; Table S1).

#### 3.1.4 Collection Sites and Author Affiliations

**Figure 3:**
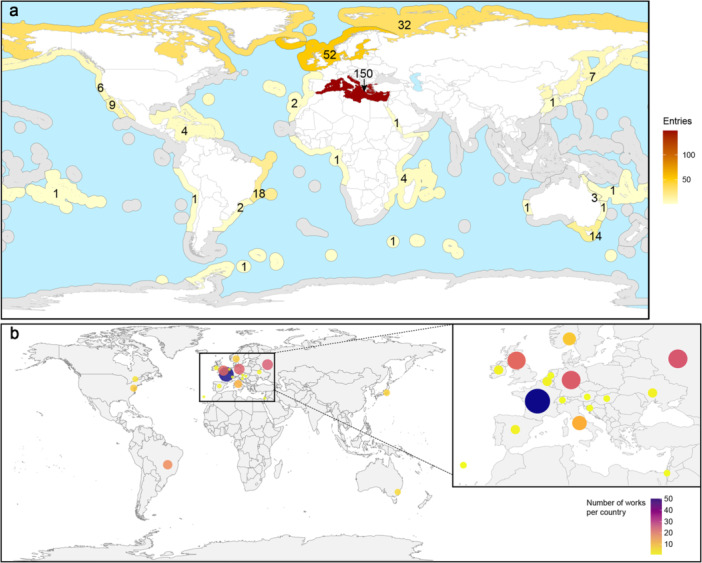
Geographical distribution of the studies on Calcarea reproduction and development. (a) Number of entries studied in each of the 62 marine provinces (MEOW) worldwide, based on literature records cross‐referenced with the World Porifera Database. (b) Map of researcher addresses per country or province, with each work counted once to indicate the institutional or regional research output. Dots indicate institutional locations and are positioned at national capitals for visualization purposes (except for Madeira Islands, Portugal).

The distribution of studied sponges reveals a pronounced geographic imbalance. Although specimens have been reported from multiple regions worldwide, sampling was concentrated in a few marine provinces. We investigated the distribution of the studies considering the Marine Ecoregions of the World (MEOW) framework (Spalding et al. 2007). The Mediterranean was the most intensively explored province, accounting for nearly half of all records (150 entries; 45.0%). Fewer records were reported from the Northern European Seas (52 entries) and the Arctic (32). Beyond these three provinces, data were sparse, with 78 entries distributed across 20 provinces, and no records available from 37 of 62 provinces (Figure 3a). This underscores large unexplored areas where Calcarea development remains undocumented. An additional 25 entries could not be confidently assigned to any specific province. However, based on the authors' affiliations and the localities of other studied populations, many are likely to have originated in provinces already investigated (Tables S1 and S3). Notably, most contributions stem from European institutions, reflecting both historical traditions and unequal current research capacity (Figure 3b).

These data indicate biases arising from uneven exploration and description efforts across the oceans. The highest species diversity of Calcarea is reported for the Warm Temperate Northwest Pacific and Southeast Australian Shelf provinces (Van Soest et al. 2012). However, both provinces had relatively few studies focusing on the reproduction or development of Calcarea. By contrast, the next most diverse provinces—the Northern European Seas and the Mediterranean Sea (Figure 3a)—partially overlap with areas where developmental data are available. Yet, none of them are in tropical regions, where biodiversity is expected to be higher. Remarkably, the five tropical marine realms (Spalding et al. 2007) host fewer described Calcarea than temperate regions, unlike Demospongiae (Van Soest et al. 2012), highlighting the limited exploration of this group in South America, the Caribbean, Africa, Southwest Asia, and the Pacific Islands. These imbalances constitute a major obstacle to advancing SRS, impeding progress in population genetics, comparative developmental biology, and the broader understanding of Calcarea evolution, reproduction, and diversity. Consequently, our current knowledge of reproduction and development is unevenly distributed across the class, being concentrated in a small number of species and study systems. Nonetheless, the nearly 200 years of investigations allow us to have some knowledge about the different steps of reproduction in Calcarea. The following sections synthesize the available evidence and highlight both the advances achieved and the gaps that persist in our understanding of reproductive and developmental processes in the group.

### 3.2 Progress and Knowledge on the Reproduction and Development of Calcarea

The reproduction in the class Calcarea can be asexual and sexual. Asexual reproduction is primarily done in two ways: the first, more common, occurs in tubular sponges and can be considered a form of budding, although it is different from the budding observed in demosponges (e.g., Connes 1964; Vasconcellos et al. 2023). Budding in tubular calcareous sponges (e.g., *Sycon*) begins with the appearance of a lateral outgrowth. This outgrowth increases in size and forms a bud that shares the aquiferous system with the parental sponge. In the following days, the aquiferous system gradually closes until the bud becomes completely independent of the parental sponge. This bud can detach and form a new individual, or it can remain attached and use the parental sponge as a substrate (Connes 1964; Tuzet 1973; Borojevic and Peixinho 1976). The second form of asexual reproduction is known as whole‐body regeneration (WBR). The ability to generate an olynthus from somatic cells isolated by passing the sponge through a mesh has been experimentally demonstrated in several studies (e.g., Huxley 1921a; Korotkova 1961, 1962, 1963a, 1963b; Gaino and Magnino 1999; Lanna and Klautau 2019, but see Ereskovsky and Lavrov (2026) for a different opinion on this subject). These findings provide direct experimental evidence that calcareous sponges can reproduce and grow through fragmentation.

Reproductive timing in calcareous sponges generally occurs during a defined seasonal period, although patterns vary among species and regions (Lanna et al. 2018b). Johnson (1978) reported a strong correlation between the reproduction of different species of Calcinea and temperature in temperate waters, whereas Van Koolwijk (1982) detected no correlation in a Calcaronea species studied in temperate regions. More recently, it was observed that in the tropical region, Calcarea appears capable of continuous reproduction, at least in *Sycettusa hastifera*, *P. magna*, and *Heteropia glomerosa* (Lanna et al. 2015; 2018; Calazans and Lanna 2019). Within the same environment, species with different life strategies can coexist, and in relatively stable regions, reproduction seems to be influenced by different environmental stimuli (Maldonado and Riesgo 2008; Lanna et al. 2018b). However, to date, most evidence linking reproduction to temperature (and other environmental factors) comes from Demospongiae, highlighting a significant gap in understanding environmental controls on sponge reproduction across other classes, particularly under current climate change scenarios.

Studies on the sexual reproduction of calcareous sponges began with the classical German‐written works of Lieberkühn (1859), Schmidt (1866), Miklucho‐Maclay (1868), and the notable contributions of Haeckel (1871), including his monograph *Die Kalkschwämme* (“The calcareous sponges*”,* 1872). It was through studying the development of calcareous sponges that he formulated his ideas about animal evolution from a gastrula: the “Gastraea theory” (Haeckel 1874). While the idea that “ontogeny recapitulates phylogeny” sparked considerable debate in biology as a whole, it also stimulated extensive research on Calcarea reproduction during the late 19th and early 20th centuries. Aligned with the Darwinian theory of descent, this perspective frames Metazoa as emerging from a single, ancient sponge‐like embryo (Reynolds 2019). In the next sessions, we will provide a historical perspective on the accumulation of knowledge that led to the current description of the reproduction and development of calcareous sponges.

#### 3.2.1 Reproduction and Development in the Subclass Calcinea

Most knowledge on the development of Calcinea dates back to the late 19th century (Haeckel 1872; Barrois 1876; Schmidt 1877; Metschnikoff 1879; Minchin 1900a), with the first illustrations and attempts to describe reproductive elements. Later, Hadži (1917) described the body growth, gametogenesis, and the larva (“spongule”) of *C. blanca*. The development of oocytes in embryos and larvae was studied in *C. coriacea* (Tuzet 1948; Sarà 1955a, 1955b), and these two *Clathrina* spp. were further examined by Johnson (1979a, 1979b), all of them in light microscopy studies. The development and metamorphosis of calciblastula larvae in six calcinean species were later studied through both light and electron microscopy (Borojevic 1969). More recent investigations have addressed oogenesis in *Clathrina aurea*, *Borojevia aspina*, and *Borojevia brasiliensis* (Lanna and Klautau 2016); embryogenesis and larval development in *Clathrina arnesenae* (Ereskovsky and Willenz 2008); the free‐swimming larvae and metamorphosis in *Soleneiscus* sp. (Amano and Hori 2001); reproduction in *Leucetta giribeti* (Riesgo et al. 2018); and regeneration after fragmentation in *C. aurea* and *Neoernsta citrea* (Padua et al. 2016; Padua and Klautau 2016). Despite these contributions, Calcinea remains a largely overlooked subclass of the SRS (Table S3).

In Calcinea, oocytes are derived from choanocytes or hyaline amoebocytes and develop within the parental mesohyl, which is likely the ancestral condition in Calcarea (Lanna and Klautau 2016), or within nests (e.g., *Ascandra* spp. and *Robspongia minchini*, Borojevic 1969), a characteristic observed only in this lineage. Although the taxonomic distribution of this character remains poorly documented, its apparent restriction to Dendyidae raises the possibility that oocyte nests constitute a lineage‐specific reproductive innovation and could provide useful information for higher‐level systematics in Calcarea. In Calcinea, the quantity of oocytes produced is often elevated, particularly in species that form nests of follicles around the oocytes, though with notable exceptions (Lanna et al. 2015; Lanna and Klautau 2016). Vitellogenesis can occur via heterosynthesis or a mixed pathway (including autosynthesis). In heterosynthesis, the oogonium grows between the choanoderm and the pinacoderm, projecting several pseudopodia, during which they phagocytose nourishing cells (nurse cells) to support growth; however, the biochemical and molecular composition of yolk in this subclass (as for the other Porifera lineages) remains largely uncharacterized (Sarà 1955a; Lanna and Klautau 2016). At the end of oogenesis, the oocyte shows a large nucleolated nucleus surrounded by granular cytoplasm.

**Figure 4:**
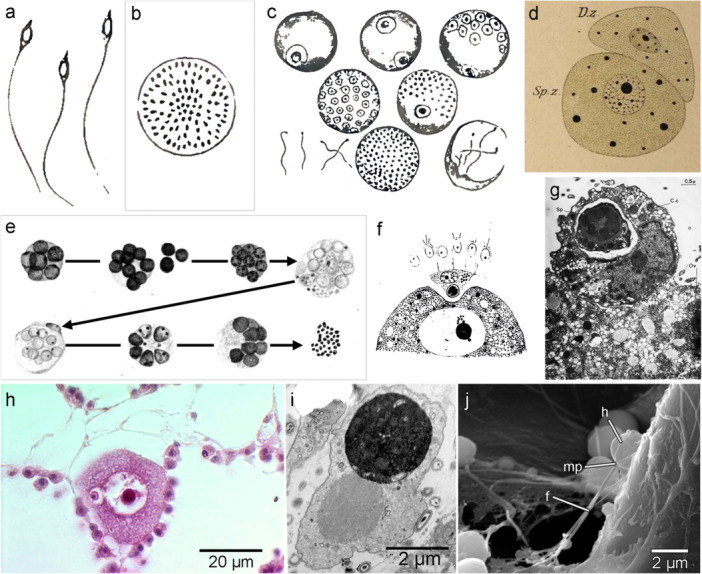
Representative historical and modern examples of male reproductive elements (spermatogenesis) in Calcarea. Panels (a–f) show classical light microscopy illustrations reflecting early interpretations of spermatogenesis, while panels (g–j) illustrate ultrastructural and modern observations. Terminology follows the original authors and reflects historical interpretations. (a) “Three individual sperm cells” of *Sycon quadrangulatum* (Haeckel 1872, t. 48). (b) “A spermospore with ripe spermatozoa” of *Ascaltis poterium* (Poléjaeff 1883, t. 3). (c) “Development of spermatozoa” of *Sycon raphanus* (Minchin 1900b, after Poléjaeff, f. 55). (d) “Spermatocyte (cover cells)” of *S. raphanus* (Görich 1904, T. 31). (e) “Sperm‐morula” of *Grantia compressa* (Dendy 1914, p. 26). (f) “Transmission from sperm cyst to oocyte by the carrier cell” of *S. raphanus* (Duboscq and Tuzet 1937b, p. 7). (g) “Carrier cell and spermiocyst” of *Grantia compressa* (Gallissian 1980; reproduced by permission of Taylor & Francis group). (h) Fertilization in *Paraleucilla magna*. (i) “Spermatozoon with a compact nucleus and the granulated dense body fully formed” of *Paraleucilla magna*. (j) “A sperm cell” of *Leucetta giribeti* (Courtesy of Ana Riesgo).

If oocyte production in Calcinea is little known, the processes underlying male gamete production are even less well investigated (Ereskovsky 2010; Lanna and Klautau 2016). Spermatic cysts have never been observed in this subclass. Hadži (1917) illustrated putative sperm cells surrounding freely floating cytophores (residual cytoplasmic masses) of *C. blanca*. Bidder (1920) described dissociated cells he referred to as spermatozoa, allegedly resembling the “minute wandering cells” of Minchin. However, it was only a century later that sperm cells were identified and photographed in dismantled choanocyte chambers of the polar species *L. giribeti* (Riesgo et al. 2018), exhibiting a mid‐piece and a long flagellum, the first flagellated sperm ever photographed in Calcarea (Figure 4j). Interestingly, the morphology of this cell is highly similar to that of cnidarian sperm (Riesgo et al. 2018). Fertilization occurs via carrier cells (“cellule charriante”), and the subsequent cleavages are equal and holoblastic, following a polyaxial cleavage pattern (Borojevic 1969; Ereskovsky and Willenz 2008).

**Figure 5:**
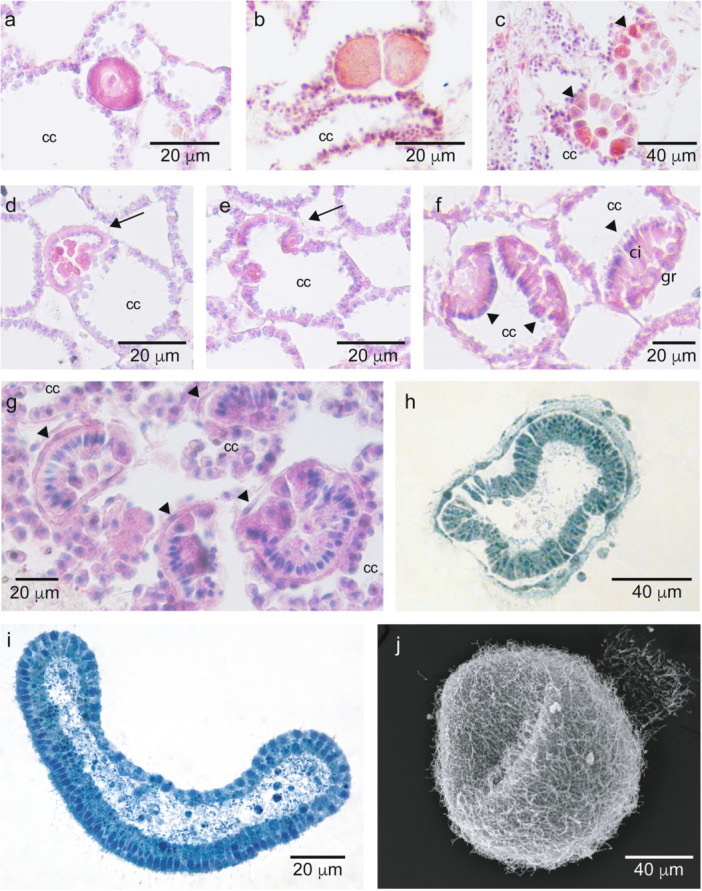
Photomicrographs of female reproductive elements and early developmental stages of Calcarea. (a–f) Calcaronea: *Heteropia glomerosa*. (a) Oocyte; (b) First cleavage; (c) Blastula cells of two embryos (*arrowheads*); (d) Mature embryo (stomoblastula) before inversion (*arrow*); (e) Stomoblastula during inversion (*arrow*); (f) Amphiblastula larvae (*arrowheads*) showing ciliated (ci) and granular cells (gr). (g) Amphiblastula larvae with an epilarval trophocyte epithelium (*arrowheads*) in the calcaronean *Leucandra serrata*. (h–j) Calcinea: *Ascaltis reticulum*. (h) Embryo; (i) Calciblastula larva. (j) Calciblastula larva. (a–g) H&E staining; (h, i) toluidine blue; (j) scanning electron microscopy. cc, choanocyte chamber; ci, ciliated cells; gr, granular cells. Panels (h–j) courtesy of Alexander Ereskovsky.

The embryo develops within a follicle composed of flattened cells, with an external layer of dense extracellular matrix and an internal layer of large cells. Symbiotic bacteria are present from this stage and persist in the larvae (Ereskovsky and Willenz 2008). The embryo gives rise to calciblastula larvae, as observed in *Ascaltis reticulum*, *Soleneiscus* sp., *C. arnesenae*, and *L. giribeti* (Amano and Hori 2001; Ereskovsky and Willenz 2008; Riesgo et al. 2018) (Figure 5h–j). Calciblastula is a hollow larva with an epithelium surrounding a large cavity. This epithelium comprises mainly two cell types: the more abundant ciliated cells, which form the entirely ciliated epithelium, and one to four granular cells located in the posterior region (Borojevic 1969; Ereskovsky and Willenz 2008) (Figure 5j). In addition to the granular cells, at least in *Soleneiscus* sp., bottle‐cells are found among the ciliated cells. These bottle‐cells lack flagella, have a basal nucleus, and a large lipid droplet in the outer portion of the cell. In addition, the vacuolar cells, located in the posterior region, are filled with large vacuoles (Amano and Hori 2001). The role of these cells is unknown, but the ultrastructural characteristics of the bottle‐cell are, somehow, similar to the cross‐cell of Calcaronea (see below).

The larva is released from the mesohyl with the water flow. After a short swimming period, some cells progressively migrate from the larval epithelium into the larval cavity, partially or completely occluding it. Although it has been suggested that this migration could be a type of gastrulation (reviewed in Leys 2004), it is more likely that these cells have a maternal origin contributing to the nourishment of the free‐swimming larvae (Amano and Hori 2001). The larva completes its planktonic phase when it swims toward the substrate and starts metamorphosis. Larval cells undergo minimal changes during this attachment, but when metamorphosis starts, they progressively change. When the larva touches the substratum, it rounds up, while the ciliated cells seem to resorb their cilia. Then, an initial differentiation takes place: ciliated cells will become pinacocytes, spreading the larva onto the substrate, while other cells become the inner cell mass. From the inner cell mass, the first choanocytes appear and form an asconoid aquiferous system, while some sclerocytes start to secrete the first triactine spicules. The young sponge, olynthus, is then formed and starts growing to reach the adult form (Amano and Hori 2001). The presence of ultrastructural markers (“glutinous granules”) in pinacocytes, sclerocytes, and choanocytes is an indication that these cells descend from the ciliated cells of the larva. The fate of bottle‐cells and granular cells is unknown, as they are not detected in the olynthus (probably being degraded during the metamorphosis), but the vacuolar cells are present in the mesohyl of the young sponge (Borojevic 1969; Amano and Hori 2001).

#### 3.2.2 Reproduction and Development in the Subclass Calcaronea

The reproduction of Calcaronea was extensively studied at the end of the 19th and the beginning of the 20th century, with recent investigations incorporating molecular approaches and species from still overlooked regions (Table S1). Consequently, we know more about the reproduction and development in this subclass than in Calcinea. In this subsection, we provide a historical perspective on the studies, followed by a thorough review of the main steps in the development of these sponges. Historical interest in this subclass was largely driven by two key embryonic events: inversion and invagination. Following the first reports of Grant (1826), Schmidt (1866) reported the presence of two poles in the “ciliated egg” of *Sycon humboldti*: an anterior pole with cilia and a posterior pole with large, non‐ciliated cells, giving rise to the current term “amphiblastula” (Figure 5).

Haeckel (1872) was the first to study larval development from the beginning of the egg segmentations. According to him, the egg would divide equally up to the 16‐cell stage, at which point 11 cells would surround five internal cells. Then, an equatorial segmentation plane would appear, generating an embryo in the shape of a biconvex lens. Subsequent cleavages would give rise to a morula, with ciliated cells on the external surface and no changes on the internal surface, which he named “pluteus.” Following this, the anterior end of the larva would disappear, and a central cavity (“stomach”) would open directly into an osculum. At this stage, the larva would be free and be called a gastrula. It would adhere to the substrate on the side opposite to the osculum, and the outer cells would lose their cilia and fuse, forming a syncytium, while the internal cells would acquire cilia. When the sponge developed its first spicules, the individual would reach the olynthus stage, a fundamental state in calcareous sponges. Haeckel's (1872, 1874) descriptions and theoretical conclusions (Gastraea theory) sparked a significant debate in the scientific community and were quickly questioned, stimulating the initial phase of reproductive studies in the class Calcarea.

Two years later, Metschnikoff (1874) revised Haeckel's descriptions, identifying a cavity within the first‐described “morula” and confirming that the larva has two halves: one half ciliated, the other of granular cells, occasionally containing spicules. He proposed that the anterior ciliated portion invaginates to form the endoderm, with the osculum appearing only at later stages, and dismissed Haeckel's interpretations as inadmissible. In 1875, Schulze described the early stages of embryogenesis, suggesting that invagination occurred just before fixation to the substrate, in the non‐ciliated region of the larva, corroborated by Keller (1876). Barrois (1876) also confirmed Schulze's cleavage observations but argued that invagination took place within the maternal body through the ciliated hemisphere, producing a *Gastrula*. By 1878, however, Schulze accepted Metschnikoff's interpretation of invagination, proposing that it could occur in the young blastula, although it was only a pseudogastrulation, while true gastrulation would result from the migration of ciliated cells toward non‐ciliated cells, forming a “mouth” at the site of larval attachment.

In the following decades, a series of works laid the foundation for the knowledge of the embryogenesis of calcareous sponges that is still held today. Dendy (1890), following Balfour (1879), described the migration of columnar cells (ciliated) into the “blastocoel” during settlement. Minchin (1896, 1900a, 1900b) conducted classical studies in which he described, among other things, a third cell type and the metamorphosis of *L. variabilis* larvae. Maas (1899, 1900) observed that the axis of the first cleavages in the oocyte is the same as that of fertilization and described the cellular ensemble that forms the sponge's body after attachment to the substrate. Hammer (1906a, 1906b; 1908) confirmed the results presented in the works of Metschnikoff (1992; 1874), Schulze (1878), Minchin (1900b), and Maas (1900), providing more detailed descriptions and publishing the first photomicrographs of the “gastrula” stage from *S. raphanus*. These works concluded the first period of reproductive biology studies of Calcarea (the first SRS period; Ereskovsky 2007).

During the second period of the SRS (Ereskovsky 2007), some studies investigated reproductive cells and documented their findings with photographs. Jörgensen (1910) observed karyogamy in fertilized oocytes and noted the formation of a polarized egg in *Sycon*. Dendy (1914) meticulously studied gametogenesis in *G. compressa*, including sperm formation. Subsequent studies not only disproved his account of spermatogenesis but also, for the first time, described a carrier cell involved in fertilization and detailed the embryo's cytology (Gatenby 1920a, 1927; Gatenby and King 1929).

Studies on Calcaronea development reached its peak with the French researchers Octave Duboscq and Odette Tuzet. They published numerous articles on various aspects of cytology, gametogenesis, and embryogenesis (Duboscq and Tuzet 1932, 1933a, 1933b, 1935a, 1935b, 1936, 1937a, 1937b, 1938a, 1938b, 1939, 1941, 1942, 1944; Tuzet 1946, 1947, 1948, 1964a, 1964b, 1970; Tuzet and Pavans de Ceccatty 1952, 1953; Tuzet and Connes 1963; Tuzet and Paris 1963). These authors were the first to describe a blastula with cilia oriented toward the blastocoel, the process of embryo inversion, and the larval cross cells (“cellules en croix”). Their detailed studies on the reproduction of *S. raphanus* and *G. compressa* culminated in a comprehensive review describing each reproductive stage (Duboscq and Tuzet 1937b).

The phenomenon of regeneration in Calcarea attracted the attention of Huxley (1911, 1921a, b), who attempted to reconstitute functional bodies from dissociated cells to reconstruct a “normal” olynthus. This line of research expanded considerably in the 1960s, largely through the extensive investigations of Korotkova et al. (1961, 1962, 1963b, 1965, 1969, 1970a, 1970b; Korotkova and Gelihovskaia 1963). Since then, regeneration has remained a prolific field of study, with important contributions continuing into recent decades (e.g., Korotkova 1997; Gaino and Magnino 1999; Eerkes‐Medrano et al. 2015; Lavrov et al. 2018; Lanna and Klautau 2019; Lavrov and Ereskovsky 2022; Skorentseva et al. 2023). As poriferans are typically capable of WBR, calcareous sponges serve as suitable model systems for investigating gene expression associated with regenerative processes, as those arising from dissociated cells or tissue injury (e.g., Soubigou et al. 2020; Ereskovsky et al. 2021; Lavrov and Ereskovsky 2022).

The 1980s–1990s saw a marked increase in electron microscopy studies on Calcarea (the third period of SRS; Ereskovsky 2007), led mainly by Marie‐France Gallissian. Most studies focused on the ultrastructure of reproductive elements in *G. compressa*, *Petrobiona massiliana*, and *S. raphanus*, addressing gametogenesis and fertilization, with some also describing the amphiblastula larvae in these and other species (Gallissian 1980, 1981, 1983, 1988, 1989; Gallissian and Vacelet 1985, 1990, 1992). Ultrastructural studies of gametogenesis and fertilization were also conducted on *S. ciliatum* (Gaino et al. 1987). Using light and electron microscopy, Franzen (1988) described the entire development of *S. ciliatum*, noting that oocytes derive from choanocytes and larval development proceeds without an embryonic follicle (later called epilarval trophocyte epithelium; Lanna and Klautau 2012). Later, electron microscopy studies examined the free‐swimming and metamorphosing larvae of *Leucandra abratsbo* and *Sycon* sp. (Amano and Hori 1992, 1993), while researchers investigated nutritive and carrier cells in *Sycon calcaravis* (Nakamura et al. 1998; Watanabe and Okada 1998). A light microscopy investigation on the embryogenesis of *L. complicata* further classified the calcaronean cleavages as “table palyntomy” (Anakina 1997; Lanna and Klautau 2012). Other contributions included studies on the sperm ultrastructure and fertilization in *L. complicata* (Anakina and Drozdov 2001), diverse aspects of the reproduction in *S. coactum* (Elliot et al. 2004; Leys 2005; Eerkes‐Medrano and Leys 2006), and the first studies carried out in the Southern Atlantic, on *P. magna* (Lanna et al. 2007, 2015; Lanna and Klautau 2010, 2012, 2025). These recent studies were later expanded to understand other tropical species, such as *H. glomerosa* and *S. hastifera* (Lanna and Klautau 2018, 2022; Calazans and Lanna 2019).

At the turn of the millennium, molecular and morphological studies were combined in research on calcareous sponge development, renewing interest in their reproduction (the fourth SRS period). Developmental genes from the Homeobox, *sine oculis*, and SOX families were identified in *Sycon* and *Leucosolenia* (Manuel and Le Parco 2000; Bebenek et al. 2004; Jager et al. 2006). After the publication of the first Porifera genome (the demosponge *Amphimedon queenslandica*; Srivastava et al. 2010), a series of genomic and transcriptomic studies have been conducted on *S. ciliatum* (Fortunato et al. 2012, 2014, 2015, 2016; Leininger et al. 2014; Bråte et al. 2015; Soubigou et al. 2020; Caglar et al. 2021). To date, all molecular investigations of developmental traits in Calcarea have focused on gene expression in *Sycon* and *Leucosolenia* species (Table S1).

##### 3.2.2.1 Spermatogenesis in Calcaronea

One of the most controversial and poorly understood aspects of reproduction in calcareous sponges is spermatogenesis. Historically, some authors claim that spermatogenesis in this class has not been extensively studied (Fell 1974; Bergquist 1978; Reiswig 1983; Simpson 1984; De Vos et al. 1991; Nakamura et al. 1998). According to studies before the use of ultrastructural methods, spermatozoa were considered to originate from an amoeboid cell (archaeocyte) that divides by amitosis (direct cell division, where the nucleus divides into two without the formation of mitotic spindles), generating a cell that would give rise to gametes and another that would form the envelope of the cyst (e.g., Poléjaeff 1882, 1883; Görich 1903, 1904; Gatenby 1920b; Farkas 1929; Tuzet 1973; Sarà and Orsi 1975) (Figure 4). This envelope would serve to nourish the spermatic cyst. However, none of these authors demonstrated the cellular processes leading to the formation of spermatozoa.

Gatenby (1920a) argued that the process described by Poléjaeff (1882) likely reflected parasitic development within the sponge, a view he extended to Dendy's (1914) account of spermatogenesis. He proposed that spermatozoa originate from mesohyl amoeboid cells, which divide to form spermatic cysts, and later observed spermatocytes differentiating from choanocytes that migrate into the choanocyte chamber, lose their flagella, and divide. From these observations, he concluded that spermatogenesis in *G. compressa* occurs in both the mesohyl and choanocyte chambers (Gatenby 1927). Sarà and Orsi (1975) later reported the presence of Gatenby's cysts in *Sycon* species, though in only two of over 100 specimens, noting that the localized and infrequent cysts posed substantial observational challenges. They disagreed, however, with the hypothesis that the speed of spermatogenesis explains its scarcity in the choanosome (Duboscq and Tuzet 1937b; Sara 1974).

Mature spermatozoa have been reported free in the choanocyte chamber or penetrating a maternal choanocyte (e.g., Gatenby 1927; Duboscq and Tuzet 1937b; Franzen 1988). Ultrastructural studies also indicate that spermatogenesis produces spermatozoa from free choanocytes beneath the choanoderm (Nakamura et al. 1998; Watanabe and Okada 1998; Anakina and Drozdov 2001; Lanna and Klautau 2010; Longo et al. 2012), as also observed in the calcinean *L. giribeti* (Riesgo et al. 2018). Lanna and Klautau (2010) investigated hundreds of *P. magna* specimens to identify spermatozoa in this species. They never observed the formation of spermatic cysts and proposed that spermatogenesis occurred within the choanocyte chambers, with the transdifferentiation of hourglass choanocytes into spermatogonia. The mature sperm were of the “aberrant” type, lacking a clear mid‐piece and flagellum (Figure 4i). The authors suggest that the absence of spermatic cysts may be one explanation for the rarity of descriptions of male gametes in Calcaronea.

So far, no study has thoroughly investigated the cellular processes of spermatogenesis in Calcarea, describing all the cellular events that lead to the transformation of choanocytes into spermatozoa. Gaps remain regarding the presence of subcellular structures, such as the nuage and acrosome. These gaps hamper comparative studies across the phylum, which exhibits remarkable diversity and has long attracted attention (e.g., Cater 1874; Vasconcellos et al. 2019). Thus, spermatogenesis remains one of the major unresolved gaps in the knowledge of reproductive biology of Calcarea, and new studies focusing on the development of male gametes need to be conducted.

##### 3.2.2.2 Oogenesis in Calcaronea

Oogenesis in calcareous sponges has been comprehensively studied within the broader context of their reproductive biology. There are two hypotheses for the formation of germ cells in this subclass. The first is that they originate from mesohyl cells, and the second is from choanocytes. Dendy (1914) unified the two hypotheses by stating that the cells that would give rise to oocytes (oogonia) were mesohyl cells that originated from choanocytes in the choanocyte chambers. This view was later supported by other researchers (Jorgensen 1917; Gatenby 1920a). Duboscq and Tuzet (1937b, 1942) also adopted this view, and in the 1980s, the passage of oogenesis through an oogonia stage (derived from choanocytes) was corroborated by electron microscopy studies (e.g., Gallissian 1981; Gaino et al. 1987; Franzen 1988). During the oogonia phase, the oocyte undergoes its first mitotic and meiotic divisions, while the cells migrate toward choanocyte chambers and transform into young oocytes (Duboscq and Tuzet 1937b). Oocyte growth was divided into two phases: “small increase” and “large increase” (Duboscq and Tuzet 1937b). The first is characterized by an amoeboid cell shape with cytoplasm poor in inclusions. Its nutrition is carried out through interdigitations between its pseudopods and choanocytes, which lose their collars and flagella. During the large increase, the oocyte is characterized by being a large, ovoid cell, usually associated with a nutritive complex, and has a large quantity of cytoplasmic inclusions (Gallissian 1981). The classification system of Duboscq and Tuzet (1937b) can be renamed as pre‐vitellogenic and vitellogenic stages of oogenesis.

The nutritive complex, consisting of an oocyte, a nurse cell derived from choanocytes and filled with granules, and an associated satellite cell, was first described for *S. raphanus* (Duboscq and Tuzet 1937b), but it is not present in all calcaroneans. Throughout the growth of the oocyte, phagocytosis of bacteria, and nurse cells occur in some species, such as *Leucosolenia botryoides*, *Leuconia nivea*, *S. elegans*, *S. ciliatum*, *P. massiliana*, *P. magna*, and *S. hastifera* (Gatenby 1920a; Duboscq and Tuzet 1942, 1944; Franzen 1988; Gallissian and Vacelet 1992; Lanna and Klautau 2010, 2022). On the other hand, other species do not undergo phagocytosis or associate with nutritive complexes during their entire growth, as is the case with *G. compressa*, *S. raphanus*, and *S. sycandra* (Gallissian 1981). Gallissian (1981) suggested that earlier reports (based on light microscopy observations) of nurse cells being phagocytosed by the oocytes could be erroneous interpretations. She argued that the former cells are intimately associated with the oocyte but are not ingested, a fact revealed only by electron microscopy.

Dendy (1914) and Gatenby (1920a) observed that these gametes are found only in certain regions of the mother sponge's body. However, the distribution of oocytes seems to be random in tropical species (*P. magna, S. hastifera*, and *H. glomerosa*; Lanna and Klautau 2010, 2022; Calazans and Lanna 2019). Sarà and Orsi (1975) and Gallissian (1981) observed that the oocyte's location at the beginning of development directly influences the formation of the nutritive complex. The formation of this complex, in turn, will influence the polarization of the oocyte and, subsequently, the cleavages and the generation of embryo cells. Although well studied, oogenesis still raises unanswered or partially explained questions, such as the origin of gametes, the phagocytosis of nurse cells, and the polarization of the oocyte (Ereskovsky 2010; Lanna and Klautau 2016).

##### 3.2.2.3 Fertilization and Embryogenesis in Calcaronea

The first reports on the fertilization of calcareous sponges were made by Haeckel (1872). In one description, he reported that spermatozoa were attracted to the oocyte and that, upon contact, both ceased movements. However, Duboscq and Tuzet (1937b) stated that “this is a romantic narrative of the fertilization process that has never been confirmed by new observations”. Contrary to Haeckel's description, fertilization in calcareous sponges occurs uniquely and exclusively in the phylum Porifera. Instead of the spermatozoon directly reaching the oocyte, an auxiliary cell, the carrier cell, is used. Gatenby (1920a) was the first author to unravel the role of this cell in the fertilization process of *G. compressa*.

The carrier cell originates from a choanocyte, which, through the apical region of the cell, captures a spermatozoon (e.g., Watanabe and Okada 1998; Anakina and Drozdov 2001; Calazans and Lanna 2019), or the spermatozoon actively enters the choanocyte (Reiswig 1983). In calcaroneans, choanocytes lose their collar and flagellum, enlarge, and migrate toward the oocyte, while the engulfed spermatozoon is enclosed in a vesicle and modified into a spermiocyst. The flagellum of the sperm regresses, usually disappearing, and the mitochondrial bodies and head become more distinct (Fell 1989). In *G. compressa*, carrier cells display a small nucleolar nucleus and vacuolated cytoplasm, and the spermiocyst is characterized by a spherical nucleus with an electron‐dense cortex and a surrounding lamellar paracrystalline body (Gallissian 1980, 1988, 1989; Reiswig 1983; Fell 1989). It was also observed that the carrier cell makes close contact with the oocyte.

One aspect of fertilization that remains poorly understood is how the spermiocyst is delivered to the oocyte. At least three possibilities exist: (i) there could be fusion between the carrier cell and the female gamete; (ii) there could be rapid binding between the two cells (a sort of cytoplasmic bridge); (iii) the carrier cell would perform exocytosis of the spermiocyst into the mesohyl, and it would penetrate the oocyte in a manner similar to that observed in other metazoans (Fell 1989). The results obtained in *G. compressa* (Duboscq and Tuzet 1937b; Gallissian 1980) support the third hypothesis; however, the extrusion was not visible in *S. calcaravis* (Watanabe and Okada 1998). The exit of the spermiocyst from its envelope activates the completion of oocyte meiosis. During the maturation division, a polar body is emitted, which is observed at the periphery of the oocyte. The spermiocyst may wait for some time until meiotic division is completed, after which it decondenses to form the male pronucleus. Then, the female pronucleus is formed, and both pronuclei fuse to form the zygote (see Tuzet 1973).

The first two cleavages in Calcaronea are total and approximately equal, but may be regular or asynchronous. However, the interpretation of the third cleavage has been approached in two different ways. The older view, adopted throughout the twentieth century (reviewed in Leys and Ereskovsky 2006), holds that after the third cleavage, the embryo consists of eight cells, all arranged laterally, forming a disc. Subsequently, the embryo would undergo radial cleavage in all cells, resulting in an embryo with 16 cells distributed in two layers. At the fifth cleavage, these two layers would undergo a second radial cleavage, generating a hollow blastula with 32 cells (16 large and 16 small). This process was initially proposed by Schulze (1875), corroborated by Duboscq and Tuzet (1937b, 1942), and later propagated by most sponge textbooks (e.g., Tuzet 1973; Fell 1974; Bergquist 1978; Simpson 1984) about sponges with radial cleavage (Ereskovsky 2010).

The other view was proposed in the late twentieth century. In a study addressing the embryogenesis of *L. complicata*, it was observed that in this species, the third cleavage is unequal and generates large and small blastomeres, with the latter being found in the furrows of the former, very similar to what is observed in the spiral cleavages of protostomes (Anakina 1997). Subsequent cleavages occur laterally, “encircling their space”, the so‐called table‐like palyntomy cleavage pattern (Anakina 1997; Eerkes‐Medrano and Leys 2006; Lanna and Klautau 2012, 2022). This results in a curved (concave) embryo, formed by a monolayer of cells and with an opening (the fialopore, or phialopore) between the granular cells. Regardless of how the blastula stage is reached, the smaller ciliated cells (micromeres) will always have a faster rate of cell division than the division of the larger granular cells (macromeres) (Leys and Ereskovsky 2006).

The cilia of the ciliated cells appear only at an advanced stage of the blastula (Duboscq and Tuzet 1937b). Initially, the cilia are oriented towards the interior of the blastocoel; that is, their orientation is the opposite of that observed in the larvae of other classes of Porifera (Leys and Ereskovsky 2006; Lanna and Klautau 2012). To correct the cilia's orientation, the embryo undergoes a dramatic conformational change. Schulze (1875) believed that the embryo cells rotate 180° to position the cilia on the outside of the larva. The reorientation of the embryo, as we know it today, was described for *S. raphanus* by Duboscq and Tuzet (1935b). As in all species studied in Calcaronea, the blastula undergoes a process of inversion, a unique and remarkable morphogenetic event among metazoans (Lanna and Klautau 2012). The first step is the opening of a pore (fialopore) between the granular cells. At this stage, the embryo is called a stomoblastula. Subsequently, ciliated cells migrate collectively (as an epithelium) towards the pole of the granular cells (Figure 5d–f), then pass through the fialopore, and finally expose the cilia to the outside of the embryo. In other words, the embryo turns inside out (Leys and Ereskovsky 2006; Lanna and Klautau 2012, 2022).

Inversion can take place in the mesohyl with posterior formation of the epilarval trophocyte epithelium (ETE; see below) or outside of any specialized structure (Lanna and Klautau 2022). Interestingly, the way in which inversion occurs appears to be determined phylogenetically (Lanna and Klautau 2022). When the embryo inverts without the formation of the ETE, the embryo's granular cells connect, through junctions, to the adjacent choanoderm and directly invert into the lumen of the choanocyte chamber (Franzen 1988) (Figure 5f). However, when the inversion occurs inside the ETE, the granular cells do not attach to any other cell of the sponge, being surrounded by the flattened cells of this epithelium concomitantly (Lanna and Klautau 2012) (Figure 5g). Independent of where the inversion takes place, before the fialopore closes, some somatic cells from the mother sponge migrate into the new cavity. These cells are amoeboid and have a nutritive role within the larva (Gallissian 1983). It should be noted that the inversion process observed in the Calcaronea subclass is very similar to that observed in *Volvox* algae (Hallmann 2006). This similarity was first observed by Duboscq and Tuzet (1937b) and was used to highlight the relationship between these algae and sponges. Some evolutionary theories of sponges and the origin of metazoans have been proposed based on this similarity (Ivanov 1971). Eerkes‐Medrano and Leys (2006) compared their results of the observed inversion in *S. coactum* with those obtained for *Volvox* and found some similarities between the two processes. However, it is currently known that these similarities have no evolutionary significance at all, being just a case of parallel evolution.

During the inversion, maternal cells rich in inclusions migrate into the blastocoel and are phagocytosed by the embryo, providing nourishment after the inversion (Lanna and Klautau 2012). Finally, the post‐inversion embryos (i.e., larvae) comprise four main cell types: ciliated cells, granular cells, cross cells, and migrating maternal cells. All these cells are not very different ultrastructurally from those found in the embryo (Gallissian and Vacelet 1992; Lanna and Klautau 2012, 2022). Some key questions remain open and can be grouped into three main areas. First, the cellular mechanisms underlying fertilization include sperm recognition by choanocytes and carrier cell attraction to the oocyte. Second, the regulation of embryonic development and morphogenesis, particularly cell type specification (e.g., cross cells), maternal involvement in inversion, and the evolutionary and mechanistic similarities of inversion processes across lineages. Third, the role of maternal contributions across generations, including potential vertical transmission of microbial symbionts and the functions of maternal cells during larval development.

##### 3.2.2.4 The Amphiblastula Larva

The amphiblastula is the most observed and described element of the reproductive biology of the class Calcarea (Figure 2). Indeed, this type of larva has been found in one of the oldest fossil records of sponges (Li et al. 1998) and is one of the few records of fossilized cellular material belonging to the class Calcarea (see Pickett 2002). The amphiblastula is formed by a single‐layered epithelium that does not possess perceptible intercellular junctions. This epithelium is composed of three cell types: (i) ciliated cells, found at the anterior pole of the larva; (ii) granular cells, large cells that lack cilia and are found at the posterior pole of the larva; and (iii) cross cells, which are four distinct translucent cells in the equator of the larva (Maldonado and Bergquist 2002; Lanna and Klautau 2012). Inside the larval cavity, various amoeboid cells are commonly observed (Duboscq and Tuzet 1937b; Spek 1938; Lufty 1957a, 1957b; Gallissian 1983; Amano and Hori 1992; Gallissian and Vacelet 1992; Eerkes‐Medrano and Leys 2006; Ereskovsky 2010; Lanna and Klautau 2012, 2022).

The ciliated cells are polarized, columnar cells with the cilium emerging from a groove in the plasma membrane. The cilium may have a basal body with one or two accessory centrioles and two or three striated ciliary roots. The nucleolated nucleus and Golgi complex are found in the apical region of the cell, usually associated with the ciliary complex. The basal region of the cell is filled with pigment inclusions (Spek 1938; Amano and Hori 1992). The granular cells are oval‐shaped, with cytoplasm lacking obvious polarity. They present a large nucleus with an evident nucleolus and many inclusions, probably of vitelline substances (Amano and Hori 1992; Lanna and Klautau 2012, 2022). Usually, there are four cross cells, but some larvae have five of them (Tuzet 1970; Lanna and Klautau 2012, 2022). The cross cells seem to derive from the small blastomeres at the beginning of embryogenesis (Gallissian 1983; Anakina 1997; Lanna and Klautau 2010). The apical region of these cells contains many vesicles, glycogen granules, and various inclusions. There is an aggregation of electron‐dense material between the central nucleus and the apical zone of the cell, surrounded by many vesicles, called the chromatic ring (Tuzet 1973). The basal region is densely pigmented, and some lipid droplets are also observed. These cells may have a photoreceptive role, responding to light stimuli (Duboscq and Tuzet 1937b; Elliot et al. 2004; Padua et al. 2013), or they may represent a relic structure (Gallissian and Vacelet 1992), or germ cell precursor (Lanna and Klautau 2012). Consequently, the role of cross cells remains a major gap in the knowledge about the amphiblastula of Calcaronea (Lanna and Klautau 2012).

The internal cavity of the larva is small. Generally, some amoeboid cells of larval or maternal origin are found, along with many symbiotic bacteria (Maldonado and Bergquist 2002). The cells found inside the larval cavity have been referred to by different names: archaeocytes (Gatenby 1992; 1920a), mesoblasts (Lufty 1957a), microgranular cells (Franzen 1988), yolk cells (Amano and Hori 1992), nutritive amoebocytes (Leys and Ereskovsky 2006), and maternal granular amoeboid cells (Lanna and Klautau 2012). Duboscq and Tuzet (1937b) proposed that these cells originate from choanocytes of the adjacent chamber and called them “absorbed choanocytes undergoing degeneration”. Different species may have different types of amoeboid cells in the larval cavity, with their origins not being homologous. In fact, some species do not present any amoeboid cells (e.g., *S. hastifera*, Lanna and Klautau 2022). Lufty (1957a), for example, described four types of these cells and observed that two originated from maternal choanocytes, while the other two derived from ETE cells, a hypothesis corroborated by electron microscopy (Gallissian 1983; Lanna and Klautau 2012). Macrogranular amoebocytes are believed to derive from ETE cells, whereas the origin and function of microgranular amoebocytes, as classified by Lufty (1957a) and Franzen (1988), remain uncertain (Lanna and Klautau 2012).

Apparently, the origin of ETE occurred in the LEUC II lineage of Leucosolenida (*sensu* Voigt et al. 2012) (Lanna and Klautau 2022).^1^ The history of the ETE started with Schulze (1875), who was the first to demonstrate that during cleavages, the embryo is surrounded by a capsule. He observed that the cells of this capsule were discoid and would fill with granules during embryo development. Dendy (1891) observed the same capsule in a species of *Grantia* and proposed a nutritive role for this membrane. Gatenby (1920a, 1920b) and Gatenby and King (1929) provided a more refined description of the capsule and determined that it would have a nutritive function, thus asserting that it would be a type of placenta. The emission of pseudopods towards the choanocytes of the mother sponge and the larva further supports the notion that the ETE would have a nutritive function (Gallissian 1983). Duboscq and Tuzet (1937b) referred to this capsule as a protective membrane, as it supposedly protected the embryo during cleavage. However, according to Lufty (1957b), the embryo's protection would be provided by the mesohyl matrix, while the ETE plays a nutritive role. More recently, Lanna and Klautau (2012) provided new ultrastructural evidence that the ETE indeed works as a nutritive structure for the larvae.

#### 3.2.3 Molecular Basis of Development in Calcarea: The *Omics* Era

In 2007, Ereskovsky wrote about our near future, and some basic unanswered questions, such as whether the signaling molecules involved in development are the same in sponges and other metazoans, and which are the “developmental genes” working in embryonic development. In the *omics* era (i.e., genomics, transcriptomics, and proteomics), molecular studies in Calcarea have unveiled an unexpectedly complex developmental toolkit, providing new insights into the cellular and molecular mechanisms of sponge morphogenesis. Genome‐wide surveys of *S. ciliatum* have identified multiple *Sox* genes with distinct and dynamic expression domains, implicating them in embryonic specification and tissue differentiation (Fortunato et al. 2012). The idea that sponges lacked *Hox* and *ParaHox* genes was challenged by Fortunato et al. (2014), who reported the presence of a *ParaHox* gene (*Cdx*) in *S. ciliatum* and *L. complicata*, along with a diverse set of *NK* homeobox genes, all expressed during embryogenesis and metamorphosis. Nevertheless, subsequent studies refuted the presence of true *Hox* or *ParaHox* genes in sponges (Pastrana et al. 2019), and according to current knowledge, Porifera, as well as Ctenophora, are animals devoid of these genes (Wanninger 2024).

Complementary studies on gene expression profiles during *S. ciliatum* development have shown that conserved signaling pathways, including the Wnt and TGF‐β families, are expressed at higher levels at the apical pole of adults and the posterior pole of larvae. Genes such as *Brachyury* (*TBXT*) and *Gata*, and germline markers *Vasa* and *Pl10*, are associated with choanoderm formation and maintenance, indicating parallels with metazoan axis specification and germ‐layer patterning (Leininger et al. 2014). Broad comparative transcriptomic surveys across all classes of Porifera further reveal extensive repertoires of adhesion molecules, cytoskeletal regulators, and developmental signaling components, underscoring that the molecular basis for cell–cell and cell–matrix interactions was already present early in animal evolution, including calcareous sponges (Riesgo et al. 2014). Regulatory layers are also implicated: long noncoding RNAs (lincRNAs) display dynamic developmental expression, suggesting RNA‐mediated regulatory mechanisms operating at the base of Metazoa (Bråte et al. 2015). Despite these advances, critical gaps remain that constrain functional interpretation. High‐quality and chromosome‐level genomes for most calcareous sponges are still lacking (Fortunato et al. 2015; Voigt et al. 2025), and current inferences rely heavily on a few transcriptomic and fragmented genomic assemblies, limiting insights into regulatory architecture and potential gene clustering. Moreover, although these studies have provided a good advance in the knowledge about the molecular control of development in Calcarea, even the investigations with the model *S. ciliatum* have declined in frequency in recent years.

### 3.3 The Next Years: How Can We Enhance Our Understanding of Reproduction and Development in Calcarea?

In the previous section, we presented the history of research on the reproduction and development of Calcarea, outlining how we arrived at the current state of knowledge in this field. As a consequence, we highlighted different aspects that need to be addressed in the near future. Here, we develop some of these gaps more thoroughly. Spermatogenesis, or their various cellular stages/names (as spermatozoid‐looking bodies, spermospores, spermatozoa, zoosperm, spermiocyst, sperm) were presented in 45 entries, including at least 35 studies with figures of the inquired male gamete (Figure 2). However, many of the described cells are probably not sperm cells but other modified flagellated cells, such as choanocytes. Flagellated sperm have only been described in one Calcinea species (Riesgo et al. 2018), and evidence of transformed choanocyte chambers with putative male gametes has been hypothesized but remains scarce (Lanna and Klautau 2010; Gilis et al. 2011; Longo et al. 2012). This is true even for species that have been deeply investigated, with hundreds, even thousands, of specimens analyzed (e.g., *S. ciliatum* and *P. magna*). Although parthenogenesis might be present in some species, it is unlikely to be a rule, as the presence of spermiocyst is detected in many different lineages and studies with population genetics indicate a very low incidence of clones and homozygotes in the populations (e.g., Padua et al. 2018; Cavalcanti et al. 2020). Therefore, the existence of the spermatozoa is very likely. We refer to this unresolved state as the “ghost sperm” problem, as the morphological detection of this gamete is still contentious. The “ghost sperm” highlights how much remains unknown about the reproductive biology of Calcarea, a gap that contrasts with the high rates of female gamete production, the formation of new individuals, and their subsequent growth. Expanding the number of species collected and analyzed could help clarify fundamental aspects of spermatogenesis in Calcarea, including sperm entry into carrier cells, the presence of spermatic cysts in some species, and the occurrence of flagellated sperm in Calcaronea. These characteristics are likely different among the lineages, providing valuable data for the systematics of the group. Furthermore, molecular approaches could reveal whether Calcarea possesses specific markers related to spermatogenesis pathways and determine when these genes are expressed during development, allowing comparisons with the cellular components present at those stages. Therefore, a deeper understanding of spermatogenesis in Calcarea will provide crucial insights into the origin, diversification, and evolution of reproductive strategies in sponges and other early‐branching metazoans.

Fertilization in Calcarea differs from that in most metazoans. All viviparous sponges can be considered to present the “spermcast mating” (Bishop 2006), in which diluted spermatozoa released in the water are accumulated by filtering the water by the “mother” before sperm and egg come into contact. What is different in calcareous sponges is that an intermediary cell takes up the sperm from the water and delivers it to the oocyte in the sponge's mesohyl. Although it is considered a characteristic of sponges in general, carrier cells were observed only in Calcarea. Studies in Calcinea (e.g., Hadži 1917; Lanna and Klautau 2016) and in Calcaronea (e.g., Minchin 1900b; Görich 1904; Duboscq and Tuzet 1937b; Gallissian 1980; Anakina and Drozdov 2001; Lanna and Klautau 2012) have reported the presence of carrier cells (or analogous cells) containing captured sperm, forming spermiocysts and transporting them to the oocyte for fertilization (Figure 4). In our view, investigations on the gene expression profile during this stage will be useful for answering these questions. Moreover, searching for genes related to mating and sperm‐oocyte recognition will be important for understanding the relationship between sperm, the carrier cell, and oocytes.

The unique process of inversion observed in Calcaronea fueled debates about whether Porifera undergoes a true gastrulation process (see Leys 2005; Ereskovsky 2010; Lanna et al. 2015). Nevertheless, all 10 recognized sponge larval types exhibit extensive morphogenesis and cell differentiation following blastula cleavage. Sogabe et al. (2019) reframed the issue: rather than asking whether sponges possess a process homologous to gastrulation, they proposed that the fundamental biology of sponge cells is distinct. Sponges maintain continuous pluripotency, whereas eumetazoans evolved gastrulation as a mechanism to generate stable and specialized cell lineages (Sogabe et al. 2019). The inversion observed in Calcaronea shows no phylogenetic link to *Volvox*; it may therefore represent either an evolutionary innovation unique to this subclass or an ancestral mode of gastrulation conserved in Calcaronea but lost in all other sponges. Despite these intrinsic differences, calcareous sponge larvae are fully functional and have persisted for at least 500 million years, and they continue to occur today across all oceans. Even though this topic seems closed to debate, the inversion appears to be an interesting model for understanding the evolution of epithelial morphogenesis in animals. The modifications in the cytoskeleton that take part during this process, the changes in the cell “junctions,” and the forces driving the movements of the cell to turn the embryo inside out can bring new insights into the evolution of calcareous sponges (as there are three different ways in which inversion can occur, see Lanna and Klautau 2022).

Another interesting research area that should be investigated in the future is the fate of the cells of the larvae of calcareous sponges, especially the amphiblastula. It is assumed that granular cells give rise to the pinacocytes, while the ciliated cells become internal cells of the sponge (mainly choanocytes, amoeboid cells of the mesohyl, and sclerocytes). The amoeboid cells seem to be consumed by the larvae. It will be very important to follow the fate of these cells using vital markers (e.g., DiI) to confirm these past observations. However, the function and fate of cross cells are much more challenging. There are some hypotheses on the function of these cells during development, but their activity as a larval photoreceptor is the most likely (Duboscq and Tuzet 1937b; Amano and Hori 1992; Elliot et al. 2004; Padua et al. 2013). Nonetheless, the expression of different germline gene markers (Leininger et al. 2014) and dense bodies (*md3*) in the ultrastructure of these cells (Lanna and Klautau 2012) could support a role as primordial germ cells segregated early in development. These hypotheses could be tested using different experimental approaches.

Addressing the remaining gaps in our understanding of Calcarea development will require the integration of molecular, genomic, and cellular approaches. Although relatively few studies have focused on modern developmental biology in this group, several genomic resources are now emerging. Currently available transcriptomic resources for Calcarea include datasets from *Clathrina coriacea*, *Leucetta giribeti*, *Leucosolenia complicata*, *Sycon ciliatum*, *Sycon coactum*, and *Sycon capricorn* (e.g., Fortunato et al. 2014; Leininger et al. 2014; Riesgo et al. 2014; Soubigou et al. 2020; Caglar et al. 2021; Pan et al. 2026; Rossi et al. 2026). In addition to the annotated genome of *S. ciliatum*, recently deposited whole‐genome shotgun (WGS) assemblies of the calcineans *Nicola tetela* (GenBank: GCA_028565695.1) and*Clathrina lutea* (GenBank: GCA_028571005.1) provide new opportunities to investigate developmental genes and perform comparative genomic analyses. These unpublished genome assemblies are available as draft genome resources and currently lack gene annotations, which limits their immediate use for gene‐centered analyses, delaying the full inclusion of Calcarea into the *omics* era of biology. For comparison, the *S. ciliatum* genome is assembled at the chromosomal level (*n* = 13), while the *N. tetela* and *C. lutea* have 189,392 and 232,709 scaffolds, respectively (accessed in June 2026). It is interesting to note that while there is accumulated knowledge about the reproduction and development of *S. ciliatum*, the opposite occurs with the calcinean *N. tetela* and *C. lutea*. Therefore, it will be very important to investigate the reproduction and development of these sponges that already have their “genes” available to be studied. The next round of *omics* sequencing should take into account the biological knowledge accumulated for the investigated species. We believe that new data on the gene content and organization in the genomes, in addition to the gene expression profiles during the development of species present in different lineages of Calcarea, together with detailed comparative histological and ultrastructural analyses of gametogenesis and embryogenesis, will be relevant to understanding the evolution of development in this class, in sponges, and metazoans as a whole.

Regeneration studies in *S. ciliatum* have shown that postlarval developmental programs can be partially reactivated, with rapid activation of Wnt and TGF‐β signaling pathways during wound healing (Soubigou et al. 2020; Caglar et al. 2021). These findings suggest that the same signaling pathways driving embryogenesis may also operate in regeneration, underscoring the developmental plasticity of Calcarea. Although causal links are yet to be demonstrated, direct functional tests of integrins, laminins, or cytoskeletal dynamics in morphogenesis remain lacking. Bridging this gap will require the application of single‐cell transcriptomics, live imaging, and targeted perturbations (e.g., RNAi or CRISPR) to dissect how gene regulatory networks control cell differentiation, tissue remodeling, and axis formation.

Finally, as mentioned throughout this work, more knowledge is needed across different taxa from different parts of the world. This knowledge does not need to be as technological as a genome sequencing project. Basic knowledge, such as when species reproduce, how the different stages of development occur, and how the larva behaves, settles, and becomes a functional sponge, is still very important to advancing the SRS. At the population level, advancing our understanding of Calcarea dynamics is equally important, as it offers insights into gene flow, reproductive success, and resilience under environmental change. Such knowledge would not only inform conservation strategies but also illuminate evolutionary processes and clarify the ecological roles of these sponges in marine ecosystems.

In the past decade (2015–2024), 27 primary literature have addressed fragmentation and regeneration experiments, the description of reproductive elements with electron microscopy, integrative taxonomy incorporating reproductive data, and emerging *omics* approaches. Collectively, these studies examined 16 model species, 11 of which (68.7%) were studied for the first time regarding development during this period (Table S1). Together, they revealed an almost unexplored landscape of developmental biology in Calcarea, with approximately 1 in 10 species having any available data or citations on their development (Table S2). Looking forward, research on Calcarea reproduction is expected to expand across multiple fronts. Extending the questions raised by Ereskovsky (2007) and Lanna et al. (2018a, 2024), we outline some of the future directions:

I. Molecular and cellular level:

A. Genomic and transcriptomic approaches will uncover genes regulating gametogenesis, reproductive timing, asexual development, and cytoskeletal dynamics.
B. Comparative reproductive morphology combined with molecular tools and population genetics will further illuminate patterns of dispersal and connectivity among species.

II. Developmental and morphological level:

A. Detailed studies of larval development are needed to clarify the presence and role of the epilarval trophocyte epithelium, linking nutritional strategies with phylogenetic patterns.
B. New investigations into gastrulation in Calcarea will test whether this process represents a case of convergent evolution with other animals or is an evolutionary innovation.
C. Expanding morphological studies across additional species will enhance our knowledge of subclass‐ and order‐level diversity, particularly the distribution of nuage, stomoblastula inversion, and sperm.

III. Ecological and population level:

A. Analyses of responses to environmental stressors, including temperature, pH, and pollutants, will illuminate how reproductive success is modulated under changing conditions.
B. Quantitative modeling of growth and reproduction, combined with studies of gametogenesis dynamics and fertilization efficiency, will clarify reproductive strategies and their impact on the communities.

In the coming decades, we may achieve a comprehensive understanding of the molecular, cellular, and ecological mechanisms underlying Calcarea reproduction, unveiling the evolutionary drivers of life‐history diversity and enabling predictive models of their resilience to global environmental change.

## Author Contributions

**Bruno Cajado:** conceptualization, investigation, writing – original draft, methodology, validation, visualization, writing – review and editing, formal analysis, data curation. **Emilio Lanna:** conceptualization, investigation, funding acquisition, writing – original draft, validation, visualization, writing – review and editing, formal analysis, project administration, data curation, supervision, resources.

## Ethics Statement

The authors have nothing to report.

## Conflicts of Interest

The authors declare no conflicts of interest.

## Supporting information


Supporting File 1

Supporting File 2

Supporting File 3

Supporting File 4

Supporting File 5

Supporting File 6

## Data Availability

The data that support the findings of this study are available in the supplementary information of this article.

## Endnotes

1 This observation highlights the potential use of reproductive characteristics to help in the systematics of calcareous sponges.
