# Supplementary material for: Reproduction and Development in Calcareous Sponges: A Panorama of the Last Two Centuries
Source: Mol Reprod Dev. 2026 Jul 6;93(7):e70129. doi: 10.1002/mrd.70129 (PMC13336810; doi:10.1002/mrd.70129)
Supplement: Supplementary file 1 — Supporting File 1 [file MRD-93-e70129-s003.docx]

**Reproduction and development in calcareous sponges: a panorama of the last two centuries**

Bruno Cajado^1,2,3^ & Emilio Lanna^2,3^

¹Graduate Program in Biodiversity and Evolution, Institute of Biology, Federal University of Bahia, Salvador, Bahia, Brazil

²Instituto de Biologia, Universidade Federal da Bahia, Salvador, Bahia, Brazil

³National Institute of Science and Technology in Interdisciplinary and Transdisciplinary Studies in Ecology and Evolution (INCT IN-TREE), Salvador, Bahia, Brazil

**Legends of the Supplementary Tables**

**Supplementary Table S1.** Primary works arranged by year of publication. Columns include reference (*n* = 192), first author and last author addresses, geopolitical localities, provinces (MEOW), publication type, and language. Each row corresponds to a single species, listing subclass, genus, accepted and original species names, developmental visualization methods, general developmental information, data group (description, illustration, molecular data), and specifically presence/absence of description, histology, and molecular data. Additional columns indicate specific datasets: male gametes, oocytes, fertilization, embryos, larvae, post-larvae, reproductive cycle, asexual reproduction, regeneration, growth, and molecular data. Works that cite only an element are marked (C), and those providing descriptions and/or illustrations are marked (Y).

**Supplementary Table S2**. Systematic bibliometric data from primary entries. Information is organized by subclass, genus, and species. For each genus, the table lists the number of entries and studied species, compared to the total species in that genus, with the corresponding percentage. An additional column indicates the number of entries for each of the 87 species.

**Supplementary Table S3**. Organization of all entries by genus and species within each subclass, with corresponding reference, data group (description, illustration, molecular data), and provinces (MEOW), totaling all entries per species.

**Supplementary Table S4**. Review works arranged by year of publication. Columns include reference (*n* = 46), first and last author addresses, publication type, and language. Each row corresponds to a single species, listing subclass, genus, accepted and original species names, general developmental information, and the presence/absence of description, histology, and molecular data. Additional columns indicate the theme for each species and relevant observations. Works that cite only an element are marked (C), and those providing descriptions and/or illustrations are marked (Y).

**Supplementary Table S5**. Systematics of all genera and their number of species, together with their respective families and order. Data from the World Porifera Database, May 2025.

**Supplementary Material**. All 238 references formatted in APA style as provided in the supplementary tables (see below).

**Complete References** (formatted in APA 7th edition)

**Primary scientific publication**

Adamska, M., Degnan, B. M., Green, K., & Zwafink, C. (2011). What sponges can tell us about the evolution of developmental processes. *Zoology, 114*, 1–10.

Amano, S., & Hori, I. (1992). Metamorphosis of calcareous sponges 1. Ultrastructure of free-swimming larvae. *Invertebrate Reproduction & Development, 21*(2), 81–90.

Amano, S., & Hori, I. (1993). Metamorphosis of calcareous sponges 2. Cell rearrangement and differentiation in metamorphosis. *Invertebrate Reproduction & Development, 24*(1), 13–26.

Amano, S., & Hori, I. (2001). Metamorphosis of coeloblastula performed by multipotential larval flagellated cells in the calcareous sponge *Leucosolenia laxa*. *Biological Bulletin, 200*, 20–32.

Anakina, R. P. (1981). The embryological development of Barents Sea sponge *Leucosolenia complicata* Mont. In G. P. Korotkova (Ed.), *Morphogenesis in sponges*. Leningrad University Press, Leningrad.

Anakina, R. P. (1988). The early stages of fertilization of sponge *Leucosolenia complicata* Montagu (Calcispongia, Leucosoleniida) from the Barents Sea. In V. M. Koltun & S. D. Stepanjants (Eds.), *Porifera and Cnidaria. Modern and perspective investigations*. Zoological Institute USSR Academy of Science Press, Leningrad.

Anakina, R. P. (1989). *Experimental-morphological investigations of the embryogenesis in Barents Sea calcareous sponge Leucosolenia complicata Montagu* (Ph.D. thesis). Leningrad University.

Anakina, R. P. (1997). The cleavage specificity in embryos of the Barents Sea sponge *Leucosolenia complicata* Montagu (Calcispongiae, Calcaronea). In A. V. Ereskovsky, H. Keupp, & R. Kohring (Eds.), *Modern problems of poriferan biology* (Berliner Geowissenschaftliche Abhandlungen, 20, pp. 45–53). Berlin.

Anakina, R. P. (1999). Peculiarities of fertilization process in the sponge *Leucosolenia complicata* Montagu (Calcispongiae: Calcaronea) from the Barents Sea. *Memoirs of the Queensland Museum, 44*, 44.

Anakina, R. P., & Drozdov, A. L. (2000) Peculiarities of oogenesis in the Barents Sea sponge *Leucosolenia complicata* Montagu (Calcispongiae, Calcaronea). *Tsitologiia,* *42*, 128–135.

Anakina, R. P., & Drozdov, A. L. (2001). Gamete structure and fertilization in the Barents Sea sponge *Leucosolenia complicata*. *Russian Journal of Marine Biology, 27*, 143–150.

Anakina, R. P., & Korotkova, G. P. (1989). Spermatogenesis in *Leucosolenia complicata* Mont., Barents Sea sponge. *Ontogenesis, 20*, 77–86.

Balfour, F. M. (1879). On the morphology and systematic position of the Spongida. *Quarterly Journal of Microscopical Science, 19*, 103–109.

Barrois, M. C. (1876). Chapitre premier: Développement des éponges calcaires. *Annales des Sciences Naturelles, Zoologie et Paléontologie, 3*, 1–84.

Bebenek, I. G., Gates, R. D., Morris, J., Hartenstein, V., & Jacobs, D. K. (2004). sine oculis in basal Metazoa. *Development Genes and Evolution, 214*, 342–351.

Bianco, S. L. (1888). Notizie biologiche riguardanti specialmente il periodo di maturità sessuale degli animali del golfo di Napoli. *Mittheilungen aus der Zoologischen Station zu Neapal, 8*, 385-440.

Bidder, G. P. (1920). The fragrance of calcinean sponges and the spermatozoa of *Guancha* and *Sycon*. *Linnean Society Journal of Zoology, 34*, 299–327.

Borojevic, R. (1969). Étude du développement et de la différenciation cellulaire d'éponges calcaires calcinéennes (genres *Clathrina* et *Ascandra*). *Annales d'Embryologie et de Morphogenèse, 2*(1), 15–36.

Borojevic, R., & Boury-Esnault, N. (1987). Revision of the genus *Leucilla* Haeckel, 1872 with a redescription of the type species *Leucilla amorpha* Haeckel, 1872. In W. C. Jones (Ed.), *European contributions to the taxonomy of sponges* (Vol. 1, pp. 29–40).

Borojevic, R., & Peixinho, S. (1976). Eponges calcaires du nord-nord-est du Brésil. *Bulletin du Muséum National d’Histoire Naturelle, 402*, 987–1036.

Bråte, J., Adamski, M., Neumann, R. S., Shalchian-Tabrizi, K., & Adamska, M. (2015). Regulatory RNA at the root of animals: Dynamic expression of developmental lincRNAs in the calcisponge *Sycon ciliatum*. *Proceedings of the Royal Society B: Biological Sciences, 282*(1821), 20151746.

Caglar, C., Ereskovsky, A., Laplante, M., Tokina, D., Leininger, S., Borisenko, I., … Adamska, M. (2021). Fast transcriptional activation of developmental signalling pathways during wound healing of the calcareous sponge *Sycon ciliatum*. *bioRxiv*, 2021-07.

Calazans, V. P., & Lanna, E. (2019). Influence of endogenous and exogenous factors on the reproductive output of a cryptogenic calcareous sponge. *Marine Biodiversity, 49*, 2837–2850.

Carter, H. J. (1874). On the nature of the seed-like body of *Spongilla*, on the origin of the mother cell of the spicule, and on the presence of spermatozoa in Spongida. *Annals and Magazine of Natural History, Series 4, 14*, 97–111.

Cavalcanti, F. F., Padua, A., Cunha, H., Halasz, M., Nikolić, V., Barreto, S., & Klautau, M. (2020). Population differentiation supports multiple human-mediated introductions of the transatlantic exotic sponge *Paraleucilla magna* (Porifera, Calcarea). *Hydrobiologia, 847*(17), 3571-3590.

Connes, R. (1964). Contribution de l’étude de la prolifération par voie asexuée chez le *Sycon*. *Bulletin de la Société Zoologique de France, 89*, 188–195.

Cotte, M. J. (1902). Sur quelques phénomènes dégénératifs observés chez *Sycandra raphanus*. *CR Assoc Fr Av Sci, 31*, 733–739.

De Vos, L., Rutzler, K., Boury-Esnault, N., Donadey, C., & Vacelet, J. (1991). *Atlas de morphologie des Éponges – atlas of sponge morphology*. Smithsonian Institution Press.

Dendy, A. (1890). On the pseudogastrula stage in the development of calcareous sponges. *Proceedings of the Royal Society of Victoria*, 93–101.

Dendy, A. (1891a). Studies on the comparative anatomy of sponges III. On the anatomy of *Grantia labyrinthica*, Carter, and the so-called family Teichonida. *Quarterly Journal of Microscopic Science*, *32*(125), 1–39.

Dendy, A. (1891b). A monograph of the Victorian sponges, I. The organisation and classification of the Calcarea Homocoela, with descriptions of the Victorian Species. *Transactions of the Royal Society of Victoria, 3*, 1–81.

Dendy, A. (1893). Studies on the Comparative Anatomy of Sponges. V. Observations on the Structure and Classification of the Calcarea Heterocoela. *Quarterly Journal of Microscopical Science, 35,* 159–257.

Dendy, A. (1914). Observations on the gametogenesis of *Grantia compressa*. *Quarterly Journal of Microscopic Science*, *60*(3), 313–370.

Dendy, A., & Frederick, L. M. (1924). On a collection of sponges from the Abrolhos Islands, Western Australia. *Journal of the Linnean Society of London, Zoology*, *35*, 477–519.

Duboscq, O., & Tuzet, O. (1932). Sur la fécondation de *Sycon ciliatum* Lieberkühn. *Comptes Rendus des Séances de la Société de Biologie*, *109*, 829–833.

Duboscq, O., & Tuzet, O. (1933a). Quelques structures des amphiblastules d'éponges calcaires. *Comptes Rendus des Séances de l'Académie des Sciences*, *197*, 1–3.

Duboscq, O., & Tuzet, O. (1933b). Sur l’ovogenèse et la fécondation des éponges calcaires: *Grantia compressa pennigera* Haeckel et *Sycon ciliatum* Lieberkhün. *Archives de Zoologie Expérimentale et Générale*, *73*, 45–56.

Duboscq, O., & Tuzet, O. (1935a). Un nouveau stade du développement des éponges calcaires. *Comptes Rendus de l'Académie des Sciences de Paris*, *200*, 1788–1790.

Duboscq, O., & Tuzet, O. (1935b). Sur l’accroissement des ovocytes de *Sycon raphanus* O. S.: la signification des «dolly-cells». *Archives de Zoologie Expérimentale et Générale*, *77*, 71–78.

Duboscq, O., & Tuzet, O. (1936). Les amoebocytes et les cellules germinales des éponges calcaires. *Mémoires du Musée Royal d'Histoire Naturelle de Belgique*, *3*, 209–226.

Duboscq, O., & Tuzet, O. (1937a). Fusome et cellules en croix des éponges calcaires. *Comptes Rendus des Séances de l'Académie des Sciences*, *204*, 1888–1891.

Duboscq, O., & Tuzet, O. (1937b). L'ovogenèse, la fécondation et les premiers stades du développement des éponges calcaires. *Archives de Zoologie Expérimentale et Générale*, *79*, 157–316.

Duboscq, O., & Tuzet, O. (1938a). La collerette des choanocytes chez les éponges calcaires hétérocoeles. *Comptes Rendus des Séances de la Société de Biologie*, *129*, 296–298.

Duboscq, O., & Tuzet, O. (1938b). L'origine et l'évolution des cellules en croix des éponges calcaires. In *Volume jubilaire Maurice Caullery. Travaux de la Station Zoologique de Wimereux* (Vol. 13, pp. 267–277).

Duboscq, O., & Tuzet, O. (1939). Les diverses formes des choanocytes des éponges calcaires hétérocoeles et leur signification. *Archives de Zoologie Expérimentale et Générale*, *80*, 353–388.

Duboscq, O., & Tuzet, O. (1941). Sur les cellules en croix des *Sycon* (*Sycon ciliatum* Fabr., *Sycon coronatum* Ellis et Sol., *Sycon elegans* Bower.) et leur signification. *Archives de Zoologie Expérimentale et Générale*, *81*, 151–163.

Duboscq, O., & Tuzet, O. (1942). Recherches complémentaires sur l’ovogenèse la fécondation et les premiers stades du développement des éponges calcaires. *Archives de Zoologie Expérimentale et Générale*, *81*, 395–466.

Duboscq, O., & Tuzet, O. (1944). L’ovogenèse, la fécondation et les premiers stades du développement du *Sycon elegans* Bower. *Archives de Zoologie Expérimentale et Générale*, *83*, 445–459.

Eerkes-Medrano, D. I., & Leys, S. P. (2006). Ultrastructure and embryonic development of a syconoid calcareous sponge. *Invertebrate Biology*, *125*(3), 177–194.

Eerkes-Medrano, D., Feehan, C. J., & Leys, S. P. (2015). Sponge cell aggregation: Checkpoints in development indicate a high level of organismal complexity. *Invertebrate Biology*, *134*(1), 1–18.

Elliot, G. R. D., MacDonald, T. A., & Leys, S. P. (2004). Sponge larval phototaxis: A comparative study. *Bollettino dei Musei e degli Istituti Biologici dell'Università di Genova*, *68*, 291–300.

Ereskovsky, A. V., Lavrov, A. I., Bolshakov, F. V., & Tokina, D. B. (2017). Regeneration in white sea sponge *Leucosolenia complicata* (Porifera, Calcarea). *Invertebrate Zoology*, *41*, 108–113.

Ereskovsky, A. V., & Willenz, P. (2008). Larval development in *Guancha arnesenae* (Porifera, Calcispongiae, Calcinea). *Zoomorphology*, *127*, 175–187.

Farkas, B. (1929). Beiträge zur Kenntnis des feineren Baues und der Entwicklung der Spongien. *International Congress of Zoology, 10e Congrès*, Budapest, 933–941.

Fontana, T., Condor-Lujan, B., Azevedo, F., Perez, T., & Klautau, M. (2018). Diversity and distribution patterns of calcareous sponges (subclass Calcinea) from Martinique. *Zootaxa*, *4410*(2), 331–369.

Fortunato, S., Adamski, M., Bergum, B., Guder, C., Jordal, S., Leininger, S., … Adamska, M. (2012). Genome-wide analysis of the Sox family in the calcareous sponge *Sycon ciliatum*: Multiple genes with unique expression patterns. *EvoDevo*, *3*, 1–11.

Fortunato, S., Adamski, M., & Adamska, M. (2015). Comparative analyses of developmental transcription factor repertoires in sponges reveal unexpected complexity of the earliest animals. *Marine Genomics*, *24*, 121–129.

Fortunato, S. A., Adamski, M., Ramos, O. M., Leininger, S., Liu, J., Ferrier, D. E., & Adamska, M. (2014). Calcisponges have a ParaHox gene and dynamic expression of dispersed NK homeobox genes. *Nature*, *514*(7524), 620–623.

Fortunato, S. A. V., et al. (2016). Conservation and divergence of bHLH genes in the calcisponge *Sycon ciliatum*. *EvoDevo*, *7*, 1–12.

Franzen, W. (1988). Oogenesis and larval development of *Scypha ciliata* (Porifera, Calcarea). *Zoomorphology*, *107*(6), 349–357.

Gaino, E., Bavestrello, G., Cerrano, C., & Sarà, M. (1996). Survival of the calcareous sponge *Clathrina cerebrum* (Haeckel, 1872) on a vertical cliff during the summer crisis. *Italian Journal of Zoology*, *63*(1), 41–46.

Gaino, E., Burlando, B., & Buffa, P. (1987). Ultrastructural study of oogenesis and fertilization in *Sycon ciliatum* (Porifera: Calcispongiae). *International Journal of Invertebrate Reproduction and Development*, *11*, 73–82.

Gaino, E., & Magnino, G. (1999). Dissociated cells of the calcareous sponge *Clathrina*: A model for investigating cell adhesion and cell motility in vitro. *Microscopy Research and Technique*, 44, 279–292.

Gaino, E., & Valentini, F. (1972). Nuovi aspetti nella riaggregazione di *Sycon ciliatum* (Porifera, Calcispongiae). *Bollettino dei Musei e degli Istituti Biologici dell'Università di Genova*, *40*, 75–87.

Gallissian, M.-F. (1980). Étude ultrastructurale de la fécondation chez *Grantia compressa* F. *International Journal of Invertebrate Reproduction*, *2*, 321–329.

Gallissian, M.-F. (1981). Étude ultrastructurale de l'ovogenèse chez quelques éponges calcaires (Porifera, Calcarea). *Archives de Zoologie Expérimentale et Générale*, *122*, 329–340.

Gallissian, M.-F. (1983). Étude ultrastructurale du développement embryonnaire chez *Grantia compressa* F. (Porifera, Calcarea). *Archives d'Anatomie Microscopique*, *72*(1), 59–75.

Gallissian, M.-F. (1988). Étude ultrastructurale de l'ovogenèse et de la fécondation chez *Leucilla endoumensis* (Spongiaire, Calcarea). *Comptes Rendus Hebdomadaires des Séances de l'Académie des Sciences*, *306*(3), 245–252.

Gallissian, M.-F. (1989). Le spermiokyste de *Sycon sycandra* (Porifera, Calcarea): Étude ultrastructurale. *Comptes Rendus de l’Académie des Sciences*, *309*(7), 251–258.

Gallissian, M.-F., & Vacelet, J. (1985). Ultrastructure des amoebocytes éosinophiles d'éponges calcaires calcaronées. *Comptes Rendus de l'Académie des Sciences de Paris*, *300*(3), 151–155.

Gallissian, M.-F., & Vacelet, J. (1990). Fertilization and nutrition of the oocyte in the calcified sponge *Petrobiona massiliana*. In K. Rützler (Ed.), *New perspectives in sponge biology* (pp. 175–181). Smithsonian Institution Press.

Gallissian, M.-F., & Vacelet, J. (1992). Ultrastructure of the oocyte and embryo of the calcified sponge *Petrobiona massiliana* (Porifera, Calcarea). *Zoomorphology*, *112*, 133–141.

Gatenby, J. B. (1920a). The germ-cells, fertilization, and early development of *Grantia (Sycon) compressa*. *Linnean Journal of Zoology*, *36*, 261–297.

Gatenby, J. B. (1920b). Further notes on the oogenesis and fertilization of *Grantia compressa*. *Journal of the Microscopical Society*, *40*, 277–282.

Gatenby, J. B. (1927). Further notes on the gametogenesis and fertilization of sponges. *Quarterly Journal of Microscopic Science*, *71*, 173–188.

Gatenby, J. B., & King, S. B. (1929). Note on the nutrient membrane of *Grantia amphiblastula*. *Journal of the Royal Microscopical Society*, *49*, 289–291.

Gilis, M., Gosselin, P., Dubois, P., & Willenz, P. (2011). Seasonal modifications and morphogenesis of the hypercalcified sponge *Petrobiona massiliana* (Calcarea, Calcaronea). *Invertebrate Biology*, *130*(3), 193–210.

Görich, W. (1903). Zur Kenntnis der Spermatogenese bei den Poriferen und Cölenteraten. *Zoologischer Anzeiger*, *27*, 64–70.

Görich, W. (1904). Zur Kenntniss der Spermatogenese bei den Poriferen und Coelenteraten nebst Bemerkungen über die Oogenese der ersteren. *Zeitschrift für Wissenschaftliche Zoologie*, *76*, 522–543.

Grant, R. E. (1826). Remarks on the structures of some calcareous sponges. *Edinburgh New Philosophical Journal*, *1*, 166–170.

Guardiola, M., Frotscher, J., & Uriz, M. J. (2016). High genetic diversity, phenotypic plasticity, and invasive potential of a recently introduced calcareous sponge, fast spreading across the Atlanto-Mediterranean basin. *Marine Biology*, *163*, 1–16.

Hadži, J. (1917). Rezultati bioloskih istrazivanja Jadranskog Mora. Porifera. Calcarea I. *Clathrina blanca* (Miklucho-Maclay). *Prirod Istraž Jugoslav Akad Znan Umjet Zagreb*, *9–10*, 1–164.

Haeckel, E. (1871). Über die sexuelle Fortpflanzung und das natürliche System der Schwämme. *Jena Zeitschrift*, *6*, 641–651.

Haeckel, E. (1872). *Die Kalkschwämme, eine Monographie* (Vols. 1–3). Verlag von Georg Reimer.

Haeckel, E. (1874). The Gastraea-Theory, the phylogenetic classification of the animal kingdom and the homology of the germ-lamellae. *Quarterly Journal of Microscopic Science*, *14*(55), 142–164, 223–247.

Hammer, E. (1906a). Über *Sycandra raphanus* H. *Verhandlungen der Zoologischen Gesellschaft in Leipzig*, *16*, 269–273.

Hammer, E. (1906b). Zur Kenntnis des feineren Baues und Entwicklung der Calcispongien. *Sitzungsberichte der Gesellschaft Naturforschender Freunde zu Berlin*.

Hammer, E. (1908). Neue Beiträge zur Kenntnis der Histologie und Entwicklung von *Sycon raphanus*. *Archiv für Biontologie*, *2*, 289–334.

Huxley, J. S. (1911). V. Some phenomena of regeneration in *Sycon*: With a note on the structure of its collar-cells. *Philosophical Transactions of the Royal Society of London, Series B*, *202*(282–293), 165–189.

Huxley, J. S. (1921a). Further studies on restitution-bodies and free tissue culture in *Sycon*. *Quarterly Journal of Microscopical Science*, *65*, 293–322.

Huxley, J. S. (1921b). Differences in viability in different types of regenerates from dissociated sponges, with a note on the entry of somatic cells by spermatozoa. *Biological Bulletin*, *40*(3), 127–129.

Ilan, M., & Vacelet, J. (1993). *Kebira uteoides* (Porifera, Calcarea), a recent “Pharetronid” sponge from coral reefs. *Ophelia*, *38*(2), 107–116.

Ivanov, A. V. (1971). On the reason of excurvation of the embryo in the colonial Phytomonadina and calcareous sponges. *Monitore Zoologico Italiano (N. S.)*, *5*, 1–10.

Jager, M., Quéinnec, E., Houliston, E., & Manuel, M. (2006). Expansion of the SOX gene family predated the emergence of the Bilateria. *Molecular Phylogenetics and Evolution*, *39*(2), 468–477.

Johnson, M. F. (1978a). Studies on the reproductive cycles of the calcareous sponges *Clathrina coriacea* and *C. blanca*. *Marine Biology*, *50*, 73–79.

Johnson, M. F. (1978b). Significance of life history studies of calcareous sponges for species determination. *Bulletin of Marine Science*, *28*(3), 570–574.

Johnson, M. F. (1979a). Gametogenesis and embryonic development in the calcareous sponges *Clathrina coriacea* and *Clathrina blanca* from Santa Catalina Island, California. *Bulletin of the Southern California Academy of Sciences*, *78*(3), 183–191.

Johnson, M. F. (1979b). Recruitment, growth, mortality and seasonal variations in the calcareous sponges *Clathrina coriacea* (Montagu) and *C. blanca* (Miklucho-Maclay) from Santa Catalina Island, California. In C. Levi & N. Boury-Esnault (Eds.), *Colloques internationaux du CNRS, Biologie des Spongiaires* (Vol. 291, pp. 325–334). Paris: CNRS.

Jones, W. C. (1971). Spicule formation and corrosion in recently metamorphosed *Sycon ciliatum* (O. Fabricius). In D. J. Crisp (Ed.), *Fourth European Marine Biology Symposium*. Cambridge: Cambridge University Press.

Jörgensen, M. (1910). Beiträge zur Kenntnis der Einbildung, Reifung, Befruchtung und Forschung bei Schwämmen (Syconen). *Archiv für Zellforschung*, *4*, 164–242.

Jorgensen, O. (1917). Reproduction in *Grantia compressa*. *Reports of the Dove Marine Laboratory*, *6*, 26-32.

Jorgensen, O. M. (1918). Note on the larvae of *Grantia compressa*. *Reports of the Dove Marine Laboratory, Cullercoats*, *7*, 60–61.

Keller, C. (1876). *Untersuchungen über Anatomie und Entwicklungsgeschichte einiger Spongien*. Basel: Verlag.

Klautau, M., Lopes, M. V., Tavares, G., & Pérez, T. (2022). Integrative taxonomy of calcareous sponges (Porifera: Calcarea) from Réunion Island, Indian Ocean. *Zoological Journal of the Linnean Society*, *194*(3), 671–725.

Korotkova, G. P. (1961). Regeneration and somatic embryogenesis in the calcareous sponge *Leucosolenia complicata* Ment. *Acta Biologica Academiae Scientiarum Hungaricae*, *9*, 315–334.

Korotkova, G. P. (1962). Behaviour of the calcareous sponge *Leucosolenia complicata* during regeneration. *Acta Biologica Academiae Scientiarum Hungaricae*, *13*, 1–30.

Korotkova, G. P. (1963b). Régénération et embryogénèse somatique chez les éponges calcaires du type *Sycon*. *Vestnik Leningradskogo Universiteta*, *18*, 34–47.

Korotkova, G. P., & Gelihovskaia, M. A. (1963). Recherches expérimentales sur le phénomène de polarité chez les éponges calcaires du type *Ascon*. *Cahiers de Biologie Marine*, *4*, 47–60.

Korotkova, G. P., Efremova, S. M., & Kadantseva, M. (1965). The peculiarities of the morphogenesis during the development of *Sycon lingua* from small fragments of the body. *Vestnik Leningrad State University*, *21*, 14–30.

Korotkova, G. P. (1969). Особенности морфогенеза при развитии известковой губки *Leucosolenia complicata* Mont. из небольших участков стенки тела. *Vestnik Leningradskogo Universiteta*, *15*, 15–22.

Korotkova, G. P. (1970a). Étude morphologique comparée du développement des éponges à partir de cellules dissociées. *Cahiers de Biologie Marine*, *11*, 325–354.

Korotkova, G. P. (1970b). Regeneration and somatic embryogenesis in sponges. In W. G. Fry (Ed.), *The biology of the Porifera* (Vol. 25). London: Symposium of the Zoological Society.

Korotkova, G. P. (1979). Peculiarities of somatic embryogenesis in sponges. In C. Lévi & N. Boury-Esnault (Eds.), *Biologie des Spongiaires: Sponge Biology. Colloques Internationaux du Centre National de la Recherché Scientifique* (Vol. 291, pp. 53–58). Paris: CNRS.

Korotkova, G. P. (1997). *Regeneration in animals*. St. Petersburg: St. Petersburg University Press.

Lanna, E., & Klautau, M. (2010). Oogenesis and spermatogenesis in *Paraleucilla magna* (Porifera, Calcarea). *Zoomorphology*, *129*, 249–261.

Lanna, E., & Klautau, M. (2012). Embryogenesis and larval ultrastructure in *Paraleucilla magna* (Calcarea, Calcaronea), with remarks on the epilarval trophocyte epithelium (“placental membrane”). *Zoomorphology*, *131*, 277–292.

Lanna, E., & Klautau, M. (2016). Some aspects of the oogenesis of three species of clathrinid sponges (Calcarea, Porifera). *Journal of the Marine Biological Association of the United Kingdom*, *96*, 529–539.

Lanna, E., & Klautau, M. (2018). Life history and reproductive dynamics of the cryptogenic calcareous sponge *Sycettusa hastifera* (Porifera, Calcarea) living in tropical rocky shores. *Journal of the Marine Biological Association of the United Kingdom*, *98*, 505–514.

Lanna, E., & Klautau, M. (2019). The choanoderm of *Sycettusa hastifera* (Calcarea, Porifera) is able to generate new individuals. *Invertebrate Biology*, *138*, e12262.

Lanna, E., & Klautau, M. (2022). Oogenesis and embryogenesis in a cryptogenic species of calcareous sponge (Calcaronea, Heteropiidae) in the southwestern Atlantic. *Invertebrate Biology*, *141*(2), e12375.

Lanna, E., & Riesgo, A. (2020). Sponge larvae do not swim that fast: a reply to Montgomery et al. (2019). *Journal of the Marine Biological Association of the United Kingdom*, *100*(1), 181–183.

Lanna, E., Monteiro, L. C., & Klautau, M. (2007). Life cycle of *Paraleucilla magna* Klautau, Monteiro and Borojevic, 2004 (Porifera, Calcarea). In M. R. Custódio, G. Lôbo-Hajdu, E. Hajdu, & G. Muricy (Eds.), *Porifera Research - Biodiversity, Innovation and Sustainability* (Vol. 28, pp. 413–418). Rio de Janeiro: Museu Nacional - Série Livros.

Lanna, E., Paranhos, R., Paiva, P. C., & Klautau, M. (2015). Environmental effects on the reproduction and fecundity of the introduced calcareous sponge *Paraleucilla magna* in Rio de Janeiro, Brazil. *Marine Ecology*, *36*(4), 1075–1087.

Lavrov, A. I., Bolshakov, F. V., Tokina, D. B., & Ereskovsky, A. V. (2018). Sewing wounds up: the epithelial morphogenesis as a central mechanism of calcaronean sponge regeneration. *Journal of Experimental Zoology Part B: Molecular and Developmental Evolution*, *330*, 351–371.

Lavrov, A. I., & Ereskovsky, A. V. (2022). Studying Porifera WBR using the calcareous sponges *Leucosolenia*. In *Whole-Body Regeneration: Methods and Protocols* (pp. 69–93). New York, NY: Springer US.

Lavrov, A., Ekimova, I., Schepetov, D., Koinova, A., & Ereskovsky, A. (2024). The complex case of the calcareous sponge *Leucosolenia complicata* (Porifera: Calcarea): hidden diversity in Boreal and Arctic regions with description of a new species. *Zoological Journal of the Linnean Society*, *200*(4), 876–914.

Leininger, S., Adamski, M., Bergum, B., Guder, C., Liu, J., Laplante, M., ... & Adamska, M. (2014). Developmental gene expression provides clues to relationships between sponge and eumetazoan body plans. *Nature Communications*, *5*(1), 3905.

Lendenfeld, R. Von (1885). A monograph of the Australian sponges. III. The Calcispongiae. *Proceedings of the Linnean Society of New South Wales*, *9*, 1083–1150.

Leys, S. P., & Eerkes-Medrano, D. (2005). Gastrulation in calcareous sponges: In search of Haeckel's Gastraea. *Integrative and Comparative Biology*, *45*, 342–351.

Lieberkühn, N. (1859). Neue Beitrage zur Anatomie der Spongien. *Arch Anat Physiol Wiss Med*, 353–382. Taf. IX, X, XI.

Longo, C., Pontassuglia, C., Corriero, G., & Gaino, E. (2012). Life-cycle traits of *Paraleucilla magna*, a calcareous sponge invasive in a coastal Mediterranean Basin. *PLoS ONE*, *7*(8), e42392.

Lopes, M. V., & Klautau, M. (2023). Phylogeny and revision of *Leucaltis* and *Leucettusa* (Porifera: Calcarea), with new classification proposals and description of a new type of aquiferous system. *Zoological Journal of the Linnean Society*, *194*(3), 671–725.

Lufty, R. G. (1957a). On the origin of the so-called mesoblast cells in the amphiblastula larva of calcareous sponges. *La Cellule (Belgique)*, *58*, 231–237.

Lufty, R. G. (1957b). On the placental membrane of calcareous sponges. *La Cellule (Belgique)*, *58*, 239–247.

Maas, O. (1899). Ueber Reifung und Befruchtung bei Spongien. *Anatomischer Anzeiger*, *16*.

Maas, O. (1900). Die Weiterentwicklung der Syconen nach der Metamorphose. *Zeitschrift für wissenschaftliche Zoologie*, *67*.

Maas, O. (1904). Über die Wirkung der Kalkentziehung auf die Entwicklung der Kalkschwämme. *Sitzungsber. d. Ges. f. Morph. u. Phys. München*, *20*, 4–21.

Maas, O. (1906). Über die Einwirkung karbonatfreier und kalkfreier Salzlösungen auf erwachsene Kalkschwämme und auf Entwicklungsstadien derselben. *Archiv für Entwicklungsmechanik der Organismen*, *22*(4), 581–599.

Manuel, M., & Le Parco, Y. (2000). Homeobox gene diversification in the calcareous sponge, Sycon raphanus. *Molecular Phylogenetics and Evolution*, *17*(1), 97–107.

Metschnikoff, E. (1874). Zur Entwicklungsgeschichte der Kalkschwämme. *Zeitschrift für wissenschaftliche Zoologische*, *24*, 1–14.

Metschnikoff, E. (1879). Spongiologische Studien. *Zeitschrift für Wissenschaften Zoologische*, *32*, 349–387.

Miklucho-Maclay, N. (1868). Beiträge zur Kenntniss der Spongien 1. *Jenaische Zeitschrift für Medizin und Naturwissenschaft*, *4*, 221–240.

Minchin, E. A. (1896). Note on the larva and post larval development of *Leucosolenia variabilis*, H. sp., with remarks on the development of other Asconidae. *Proceedings of the Royal Society of London*, *40*, 42–52.

Minchin, E. (1900a). Éponges calcaires. La Clathrine coriace. In: Zoologie Descriptive des Invertébrés (ed. Boutain, L. M.). Paris, Doin, I: 107–147.

Minchin, E. A. (1900b). Sponges. In: A Treatise in Zoology (ed. Lankester, E. R.). Londres, Adam and Charles Black: 1–178.

Nakamura, Y., Okada, K., & Watanabe, Y. (1998). The ultrastructure of spermatozoa and its ultrastructural change in the choanocyte of Sycon calcaravis Hozawa. In: Watanabe Y., Fusetani N. (eds) *Sponge sciences. Multidisciplinary perspectives*. Springer, Tokyo.

Orton, J. H. (1914). Preliminary account of a contribution to an evaluation of the sea. *Journal of the Marine Biological Association of the United Kingdom*, *10*(2), 312–326.

Orton, J. H. (1920). “Sea-temperature, Breeding, and Distribution in Marine Animals”. *J. Mar. Biol. Assoc. Plymouth*, *12*, 339.

Padua, A.; Klautau, M. (2016) Regeneration in calcareous sponges (Porifera). *Journal of the Marine Biological Association of the United Kingdom*, *96*(2), 553-558.

Padua, A., Lanna, E., Zilberberg, C., Paiva, P. C. D., & Klautau, M. (2013). Recruitment, habitat selection and larval photoresponse of *Paraleucilla magna* (Porifera, Calcarea) in Rio de Janeiro, Brazil. *Marine Ecology*, *34*(1), 56–61.

Padua, A., Leocorny, P., Custódio, M. R., & Klautau, M. (2016). Fragmentation, fusion, and genetic homogeneity in a calcareous sponge (Porifera, Calcarea). *Journal of Experimental Zoology Part A: Ecological Genetics and Physiology*, *325*(5), 294–303.

Padua, A., Cunha, H. A., & Klautau, M. (2018). Gene flow and differentiation in a native calcareous sponge (Porifera) with unknown dispersal phase: Population structure of *C. aurea*. *Marine Biodiversity, 48*(4), 2125-2135.

Poléjaeff, N. (1882). Über das Sperma und die Spermatogenesis bei *Sycandra raphanuz* Haeckel. *Sitzungsberichte / Akademie der Wissenschaften in Wien*, *86*, 276–298.

Poléjaeff, N. (1883). Voyage of HMS Challenger. Zoology - Vol. VIII - II.—Report on the Calcarea dredged by H.M.S. Challenger, during the years 1873–1876.

Riesgo, A., Farrar, N., Windsor, P. J., Giribet, G., & Leys, S. P. (2014a). The analysis of eight transcriptomes from all poriferan classes reveals surprising genetic complexity in sponges. *Molecular Biology and Evolution*, *31*(5), 1102–1120.

Riesgo, A., Novo, M., Sharma, P. P., Peterson, M., Maldonado, M., & Giribet, G. (2014b). Inferring the ancestral sexuality and reproductive condition in sponges (Porifera). *Zoologica Scripta*, *43*(1), 101–117.

Riesgo, A., Cavalcanti, F. F., Kenny, N. J., Ríos, P., Cristobo, J., & Lanna, E. (2018). Integrative systematics of clathrinid sponges: Morphological, reproductive and phylogenetic characterization of a new species of *Leucetta* from Antarctica (Porifera, Calcarea, Calcinea) with notes on the occurrence of flagellated sperm. *Invertebrate Systematics*, *32*, 827–841.

Sarà, M. (1955a). La nutrizione dell'ovocita in calcispongie omoceli. *Annuario dell'Instituto e Museo di Zoologia dell'Università di Napoli*, *7*, 1–30.

Sarà, M. (1955b) Sulle cellule nutrici nell'ovogenesi delle Calcispongie Omoceli, *Bolletino di zoologia, 22*(2), 323-327.

Sarà, M. (1974). Sexuality in the Porifera. *Bolletino di Zoologia*, *41*(4), 327–348.

Schmidt, O. (1866). Zweites Supplement der Spongien des Adriatischen Meeres; enthaltend die Vergleichung der Adriatischen und britischen Spongien-Gattungen. Leipzig.

Sarà, M., Gaino, E., & Valentini, F. (1974). Olynthus formation by cell aggregation in *Sycon vigilans* (Porifera, Calcispongiae). *Vie Milieu, Vol. XXIV, fasc. 2, sér. A*, 225-234.

Sarà, M., & Orsi, L. R. (1975). Sex differentiation in *Sycon* (Porifera Calcispongiae). *Pubblicazioni della Stazione Zoologica di Napoli*, *39*, 618–634.

Schmidt, O. (1875). Zur Orientirung über die Entwicklung der Spongien. *Z. wiss. Zool.*, 25, 127–141.

Schmidt, O. (1877). Das Larvenstadium von *Ascetta primordialis* und *Ascetta clathrus*. *Archiv für mikroskopische Anatomie*, *14*, 249–263.

Schulze, F. E. (1875). Über den Bau und die Entwicklung von *Sycandra raphanus* Haeckel. *Zeitschrift für wissenschaftliche Zoologische*, 25, 247–280.

Schulze, F. E. (1878). Untersuchungen über den Bau und die Entwicklung der Spongien. Die Metamorphose von *Sycandra raphanus*. *Zeitschrift für wissenschaftliche Zoologische*, *31*, 262–295.

Sebé-Pedrós, A., Ariza-Cosano, A., Weirauch, M., Leininger, S., Yang, A., Torruella, G., Adamski, M., Adamska, M., Hughes, T., Gómez-Skarmeta, J., & Ruiz-Trillo, I. (2013). Early evolution of the T-box transcription factor family. *Proc. Natl. Acad. Sci. U. S. A.*, *110*, 16050–16055.

Skorentseva, K. V., Bolshakov, F. V., Saidova, A. A., & Lavrov, A. I. (2023). Regeneration in calcareous sponge relies on ‘purse-string’ mechanism and the rearrangements of actin cytoskeleton. *Cell and Tissue Research*, *394*(1), 107–129.

Soubigou, A., Ross, E. G., Touhami, Y., Chrismas, N., & Modepalli, V. (2020). Regeneration in the sponge *Sycon ciliatum* partly mimics postlarval development. *Development*, *147*(22), dev193714.

Spek, J. (1938). Studien über die Polarität der Larven der Kalkschwämme. *Protoplasma*, *30*(1), 352–372.

Topsent, E. (1887). Contribution à l’étude des Clionides. *Archives de Zoologie Expérimentale et Générale*, *Ser. 2,* *5*(Suppl.), 1–165.

Tuzet, O. (1946). L'embryologie des éponges calcaires hétérocoeles. *Les Editions de la Revue scientifique,* *84*(7), 405–409

Tuzet, O. (1947). L'ovogenèse et la fécondation de l'éponge calcaire *Leucosolenia* (*Clathrina*) *coriacea* Mont et de l'éponge siliceuse *Reniera elegans* Bow. *Archives de Zoologie Expérimentale et Générale*, *85*, 127–148.

Tuzet, O. (1948). Les premiers stades du développement de *Leucosolenia botryoides* Ellis et Sollander et de *Clathrina* (*Leucosolenia*) *coriacea* Mont. *Annales des Sciences Naturelles, Zoologie et Biologie*, *10*, 103–114.

Tuzet, O. (1964a). Remarque sur la classification des *Sycon* telle qu'elle a été conçue par Burton (1963).

Tuzet, O. (1964b). L’origine de la lignée germinale et la gamétogenèse chez les spongiaires. In *L’Origine de la Lignée Germinale* (Ed. E. Wolff), pp. 79–111.

Tuzet, O. (1970). La polarité de l'oeuf et la symétrie de la larve des éponges calcaires. *Symposium of the Zoological Society of London*, *25*, 437–448.

Tuzet, O., & Pavans de Ceccatty, M. (1952). Les cellules de *Grantia compressa pennigera* Haeckel (Éponge Calcaire Hétérocoele). *Comptes Rendus Hebdomadaires des Séances de L'Academie des Sciences*, *235*(23), 1541–1543.

Tuzet, O., & Pavans de Ceccaty, M. (1953). Les cellules nerveuses de l'éponge calcaire homocele *Leucandra jonhstoni* Cart. *Comptes-Rendus des Séances de l'Académie des Sciences de Paris*, *236*, 130–133.

Tuzet, O., & Paris, J. (1963). Recherches sur la régénération de *Sycon raphanus* o. *S.* *Vie et Milieu*, 285–292.

Tuzet, O., & Connes, R. (1963). Recherches histologiques sur la reconstitution de *Sycon raphanus* O.S. à partir de cellules dissociées. *Vie & Milieu*, *13*(4), 703–710.

Vacelet, J. (1960). Les choanocytes et la larve de *Petrobiona massiliana* Vacelet et Lévi 1958. Éponge Pharétronide méditerranéenne. *Comptes rendus hebdomadaires des séances de l'Académie des Sciences*, *251*(3), 2405–2407.

Vacelet, J. (1964). Étude monographique de l’éponge calcaire pharétronide de Méditerranée, *Petrobiona massiliana* Vacelet et Lévi. Les pharétronides actuelles et fossiles. *Recueil des Travaux de la Station Marine d'Endoume*, *34*(50), 1–125.

Vacelet, J. (1967). Descriptions d’éponges Pharétronides actuelles des tunnels obscurs sous-récifaux de Tuléar (Madagascar). *Recueil des Travaux de la Station marine d’Endoume*, 37–62.

Vacelet, J. (1977). Éponges pharétronides actuelles et sclérosponges de Polynésie française, de Madagascar et de la Réunion. *Bulletin du Muséum national d’Histoire naturelle*, *444*, 345–366.

Vacelet, J. (1991). Recent Calcarea with a reinforced skeleton (“Pharetronids”). In *Fossil and Recent sponges* (pp. 252–265). Berlin, Heidelberg: Springer Berlin Heidelberg.

Van Koolwijk, T. (1982). Calcareous sponges of the Netherlands (Porifera, Calcarea). *Bulletin Zoologisch Museum, Universiteit van Amsterdam*, *8*, 89–98.

Vasseur, G. (1879). Reproduction asexuelle de la *Leucosolenia botryoides* (Ascandra variabilis). *Archs Zool Exp Gén*, *8*, 59–66.

Watanabe, Y., & Okada, K. (1998). The involvement of two carrier cells in fertilization and the ultrastructure of the spermiocyst in *Sycon calcaravis*. In *Sponge sciences: multidisciplinary perspectives: Proceedings of the International Conference on Sponge Science*. Tokyo: Springer-Verlag, 193–202.

Wörheide, G., & Hooper, J. N. A. (2003). New species of Calcaronea (Porifera: Calcarea) from cryptic habitats of the southern Great Barrier Reef (Heron Island and Wistari Reef, Capricorn-Bunker Group, Australia). *Journal of Natural History*, *37*, 1–47.

Yamasaki, A., & Watanabe, Y. (1991). Involvement of maternal choanocyte during embryogenesis in *Sycon calcaravis*, Calcarea, Porifera. *Zool Sci*, *8*, 1107.

**Review publications**

Batigina, T. B., Bragina, E. A., Ereskovsky, A. V., & Ostrovsky, A. N. (2006). *Viviparity in plants and invertebrate animals*. St. Petersburg University Press.

Bergquist, P. R. (1978). Reproduction and development. In *Sponges* (pp. 99–132). Berkeley and Los Angeles: University of California Press.

Borojevic, R. (1970). Différenciation cellulaires dans l’émbryogenèse et la morphogenèse chez les Spongiaires. In W. G. Fry (Ed.), *The biology of the Porifera* (Vol. 25, Symposium of the Zoological Society, London).

Borojevic, R., Boury-Esnault, N., & Vacelet, J. (1990). A revision of the supraspecific classification of the subclass Calcinea (Porifera, class Calcarea). *Bulletin du Museum National d’Histoire Naturelle, Section A, Zoologie Biologie et Ecologie Animales, 12*, 243–276.

Borojevic, R., Boury-Esnault, N., & Vacelet, J. (2000). A revision of the supraspecific classification of the subclass Calcaronea (Porifera, class Calcarea). *ZOOSYSTEMA-PARIS-, 22*(2), 203-264.

Brien, P. (1943). L'embryologie des éponges. *Bulletin du Muséum d’Histoire Naturelle de Belgique, 19*, 1–20.

Brien, P. (1967). Leur nature métazoaire, leur gastrulation, leur état colonial. *Annales de la Société Royale Zoologique de Belgique, 97*(4), 197–235.

Brien, P. (1972). Les feuillets embryonnaires des éponges. Réponse au Professeur A. V. Ivanov. *Bulletins de l'Académie Royale de Belgique, 58*(1), 715–732.

Burton, M. (1963). *A revision of the classification of the calcareous sponges: with a catalogue of the specimens in the British Museum (Natural History)*. William Clowes and Sons, London.

Degnan, B. M., Adamska, M., Richards, G. S., Larroux, C., Leininger, S., Bergum, B., … & Degnan, S. M. (2015). Porifera. In *Evolutionary developmental biology of invertebrates 1: Introduction, Non-Bilateria, Acoelomorpha, Xenoturbellida, Chaetognatha* (pp. 65–106).

Ereskovsky, A. V. (2004). Comparative embryology of sponges and its application for Poriferan phylogeny. *Bollettino dei Musei e degli Istituti Biologici dell'Università di Genova, 68*, 301–318.

Ereskovsky, A. V. (2007). Sponge embryology: The past, the present and the future. In M. R. Custódio, G. Lôbo-Hajdu, E. Hajdu, & G. Muricy (Eds.), *Porifera Research - Biodiversity, Innovation and Sustainability* (pp. 41–52). Rio de Janeiro: Museu Nacional.

Ereskovsky, A. V. (2010). *The comparative embryology of sponges*. Springer Science & Business Media.

Ereskovsky, A., Borisenko, I. E., Bolshakov, F. V., & Lavrov, A. I. (2021). Whole-body regeneration in sponges: Diversity, fine mechanisms, and future prospects. *Genes, 12*(4), 506.

Fell, P. E. (1974). Porifera. In A. C. Giese & J. S. Pearse (Eds.), *Reproduction of marine invertebrates. Volume I: Acoelomate and pseudocoelomate metazoans* (pp. 51–132). New York & London: Academic Press.

Fell, P. E. (1989). Porifera. In K. G. Adiyodi & R. G. Adiyodi (Eds.), *Reproductive biology of invertebrates. Volume IV, Part A: Fertilization, development, and parental care* (pp. 1–41). Chichester, England: John Wiley & Sons.

Fell, P. E. (1993). Porifera. In K. G. Adiyodi & R. G. Adiyodi (Eds.), *Reproductive biology of invertebrates. Volume VI, Part A: Asexual propagation and reproductive strategies* (pp. 1–44). Chichester, England: John Wiley & Sons.

Grell, K. G. (1979). Die Gastraea-Theorie. *Medizinhistorisches Journal, H. 4*, 275–291.

Harrison, F. W., & De Vos, L. (1991). Porifera. In F. W. Harrison (Ed.), *Microscopic anatomy of invertebrates* (pp. 29–89).

Hartman, W. D. (1958). A re-examination of Bidder's classification of the Calcarea. *Systematic Zoology, 7*(3), 97–110.

Ivanova-Kazas, O. M. (1975). Comparative embryology of invertebrates: Protista and lower invertebrates. Nauka, Novosibirsk.

Ivanova-Kazas, O. M. (1995). Evolutionary embryology of animals. Nauka, St. Petersburg.

Korotkova, G. P. (1963a). On the types of restoration processes in sponges. *Acta Biologica Academiae Scientiarum Hungaricae, 13*, 389–406.

Korotkova, G. P. (1981). General characteristics of sponge organization. In G. P. Korotkova (Ed.), *Morphogenesis in sponges*. Leningrad University Press.

Korotkova, G. P. (1988). Peculiarities of organization and types of the development in sponges. In V. M. Koltun & S. D. Stepanjants (Eds.), *Porifera and Cnidaria: Modern and perspective investigations*. Zoological Institute, USSR Academy of Sciences.

Lanna, E. (2015). Evo-devo of non-bilaterian animals. *Genetics and Molecular Biology, 38*, 284–300.

Lanna, E., Cajado, B., Santos, D., Cruz, F., Oliveira, F., & Vasconcellos, V. (2018). Outlook on sponge reproduction science in the last ten years: Are we far from where we should be? *Invertebrate Reproduction & Development, 62*(3), 133–142.

Lanna, E., Riesgo, A., Koutsouveli, V., & Leys, S. P. (2024). Chapter 1: Physiology of reproduction in Porifera. In S. Saleuddin, S. P. Leys, R. D. Roer, & I. C. Wilkie (Eds.), *Frontiers in invertebrate physiology, Volume 1: Non-Bilaterian phyla*. Apple Academic Press/CRC Press.

Lemche, H., & Tendal, O. S. (1977). An interpretation of the sex cells and the early development in sponges, with a note on the terms acrocoel and spongocoel. *Journal of Zoological Systematics and Evolutionary Research, 15*(4), 241–252

Levit, G. S., Hossfeld, U., Naumann, B., Lukas, P., & Olsson, L. (2022). The biogenetic law and the Gastraea theory: From Ernst Haeckel's discoveries to contemporary views. *Journal of Experimental Zoology Part B: Molecular and Developmental Evolution, 338*(1–2), 13–27.

Leys, S. (2004). Gastrulation in sponges. In C. D. Stern (Ed.), *Gastrulation: From cells to embryo* (Vol. 1, pp. 23–31). Cold Spring Harbor, NY: Cold Spring Harbor Laboratory Press.

Leys, S. P., & Ereskovsky, A. V. (2006). Embryogenesis and larval differentiation in sponges. *Canadian Journal of Zoology, 84*, 262–287.

Maldonado, M. (2004). Choanoflagellates, choanocytes, and animal multicellularity. *Invertebrate Biology, 123*(1), 1–22.

Maldonado, M. (2014). Chapter 16. Metazoans: The rise of early animals. In P. Vargas & R. Zardoya (Eds.), *The tree of life: Evolution and classification of living organisms* (pp. 182–205). Sinauer Associates.

Maldonado, M., & Bergquist, P. R. (2002). Phylum Porifera. In C. M. Young, M. A. Sewell, & M. E. Rice (Eds.), *Atlas of marine invertebrate larvae* (pp. 21–50). San Diego: Academic Press.

Maldonado, M., & Riesgo, A. (2008). Reproduction in the phylum Porifera: a synoptic overview. T*reballs de la Societat Catalana de Biologia, 59*, 29-49.

Manuel, M. (2009). Early evolution of symmetry and polarity in metazoan body plans. *Comptes Rendus Biologies, 332*(2–3), 184–209.

Manuel, M., Borchiellini, C., Alivon, E., Le Parco, Y., Vacelet, J., & Boury-Esnault, N. (2003). Phylogeny and evolution of calcareous sponges: Monophyly of Calcinea and Calcaronea, high level of morphological homoplasy, and the primitive nature of axial symmetry. *Systematic Biology, 52*(3), 311–333.

Nielsen, C. (2008). Six major steps in animal evolution: Are we derived sponge larvae? *Evolution & Development, 10*(2), 241–257.

Ostrovsky, A. N., Lidgard, S., Gordon, D. P., Schwaha, T., Genikhovich, G., & Ereskovsky, A. V. (2016). Matrotrophy and placentation in invertebrates: A new paradigm. *Biological Reviews, 91*(3), 673–711.

Reiswig, H. M. (1983). Porifera. In K. G. Adiyodi & R. G. Adiyodi (Eds.), *Reproductive biology of invertebrates. Vol. II, Spermatogenesis and sperm function* (pp. 1–21). New York: John Wiley & Sons.

Reynolds, A. S. (2019). Ernst Haeckel and the philosophy of sponges. *Theory in Biosciences, 138*, 133–146.

Simpson, T. L. (1984). *The cell biology of sponges*. New York: Springer-Verlag.

Tuzet, O. (1973). Éponges calcaires. In P.-P. Grassé (Ed.), *Traité de zoologie: Spongiaires – anatomie, physiologie, systématique, écologie* (Vol. III, pp. 27–132). Paris: Masson et Cie.

Woollacott, R. M., & Pinto, R. L. (1995). Flagellar basal apparatus and its utility in phylogenetic analyses of the Porifera. *Journal of Morphology, 226*(3), 247–265.

Wörheide, G., Dohrmann, M., Erpenbeck, D., Larroux, C., Maldonado, M., Voigt, O., … & Lavrov, D. V. (2012). Deep phylogeny and evolution of sponges (phylum Porifera). *Advances in Marine Biology, 61*, 1–78.
